# Structure of a dimeric photosystem II complex from a cyanobacterium acclimated to far-red light

**DOI:** 10.1016/j.jbc.2022.102815

**Published:** 2022-12-20

**Authors:** Christopher J. Gisriel, Gaozhong Shen, David A. Flesher, Vasily Kurashov, John H. Golbeck, Gary W. Brudvig, Muhamed Amin, Donald A. Bryant

**Affiliations:** 1 Department of Chemistry, Yale University, New Haven, Connecticut, USA; 2 Department of Biochemistry and Molecular Biology, The Pennsylvania State University, University Park, Pennsylvania, USA; 3 Department of Molecular Biophysics and Biochemistry, Yale University, New Haven, Connecticut, USA; 4 Department of Chemistry, The Pennsylvania State University, University Park, Pennsylvania, USA; 5 Department of Sciences, University College Groningen, University of Groningen, Groningen, the Netherlands; 6 Rijksuniversiteit Groningen Biomolecular Sciences and Biotechnology Institute, University of Groningen, Groningen, the Netherlands; 7 Center for Free-Electron Laser Science, Deutsches Elektronen-Synchrotron DESY, Hamburg, Germany

**Keywords:** photosynthesis, cyanobacteria, photosystem II, chlorophyll, far-red light photoacclimation, energy transfer, electron transfer, PsbH, cofactor assignment, cryo-EM, DGD, digalactosyl-diacylglycerol, FaRLiP, far-red light photoacclimation, FRL, far-red light, LHCII, light-harvesting complex II, MES, 2-(N-morpholino) ethanesulfonic acid, OEC, oxygen-evolving complex, PPB, photosystem purification buffer, PSI, photosystem I, PSII, photosystem II, VL, visible light, β-DM, β-D-maltoside

## Abstract

Photosystem II (PSII) is the water-splitting enzyme central to oxygenic photosynthesis. To drive water oxidation, light is harvested by accessory pigments, mostly chlorophyll (Chl) *a* molecules, which absorb visible light (400–700 nm). Some cyanobacteria facultatively acclimate to shaded environments by altering their photosynthetic machinery to additionally absorb far-red light (FRL, 700–800 nm), a process termed far-red light photoacclimation or FaRLiP. During far-red light photoacclimation, FRL-PSII is assembled with FRL-specific isoforms of the subunits PsbA, PsbB, PsbC, PsbD, and PsbH, and some Chl-binding sites contain Chls *d* or *f* instead of the usual Chl *a*. The structure of an apo-FRL-PSII monomer lacking the FRL-specific PsbH subunit has previously been determined, but visualization of the dimeric complex has remained elusive. Here, we report the cryo-EM structure of a dimeric FRL–PSII complex. The site assignments for Chls *d* and *f* are consistent with those assigned in the previous apo-FRL-PSII monomeric structure. All sites that bind Chl *d* or Chl *f* at high occupancy exhibit a FRL-specific interaction of the formyl moiety of the Chl *d* or Chl *f* with the protein environment, which in some cases involves a phenylalanine sidechain. The structure retains the FRL-specific PsbH2 subunit, which appears to alter the energetic landscape of FRL-PSII, redirecting energy transfer from the phycobiliprotein complex to a Chl *f* molecule bound by PsbB2 that acts as a bridge for energy transfer to the electron transfer chain. Collectively, these observations extend our previous understanding of the structure-function relationship that allows PSII to function using lower energy FRL.

Photosystem II (PSII) in oxygenic phototrophs is the multisubunit membrane protein complex that couples light absorption with water oxidation to generate reducing equivalents for carbon fixation (1, 2). A complete PSII holocomplex is typically found in a dimeric configuration, each monomer comprising about 20 subunits that bind many cofactors (3). Chlorophyll (Chl) molecules account for 35 of these cofactors, most of which serve primarily to harvest light. Upon light absorption, energy is transferred through Chl molecules until its arrival at a series of cofactors that comprise the electron transfer chain. Charge separation occurs when an electron is transferred to a plastoquinone on the acceptor side of the complex. The hole left on the donor side triggers the catalytic advancement of a Mn_4_CaO_5-6_ metallocofactor called the oxygen-evolving complex (OEC) that extracts electrons from water (4, 5).

Many characteristics and properties of PSII are highly conserved and nearly invariant among all oxygen-evolving phototrophs, but like all bioenergetic systems, some differences have evolved among species, typically due to environmental pressures within specific ecological niches (6). Whereas most oxygenic phototrophs use primarily visible light (VL, 400–700 nm) to drive PSII function, some cyanobacteria have evolved to additionally use lower energy, far-red light (FRL, 700–800 nm) for this purpose (7). The best characterized of these strategies is a facultative response mechanism to shaded environments called far-red light-photoacclimation or FaRLiP (8, 9, 10). Cyanobacteria capable of FaRLiP contain a unique gene cluster, most of which encodes proteins involved in Chl *f* biosynthesis and FRL-specific paralogs of photosystem subunits. Upon expression of the FaRLiP gene cluster, five subunits of PSII become FRL-specific: the subunits PsbA (*i.e.*, D1), PsbB (*i.e.*, CP47), PsbC (*i.e.*, CP43), PsbD (*i.e.*, D2), and also PsbH, a PSII subunit with a single transmembrane α-helix (FRL-specific isoforms of these subunits are called PsbA3, PsbB2, PsbC2, PsbD3, and PsbH2, respectively) (8, 9, 10, 11). Additionally, whereas all 35 Chls bound by PSII (per monomer) are Chl *a* when cells are grown in VL, the FRL-specific PSII complex (FRL-PSII) binds a mixture of different Chl types in those same positions: about 30 Chl *a*, 4 Chl *f*, and 1 Chl *d* (8, 9, 10, 11, 12). These FRL-specific alterations allow PSII to achieve water oxidation using lower energy FRL, decreasing the low energy limit that was canonically thought to be required to drive oxygenic photosynthesis (13).

An understanding of the molecular basis of FRL-PSII function is of great interest because it may allow for the engineering of shade tolerance into crops (14, 15). To gain the requisite understanding, various spectroscopic, phylogenetic, and structural investigations have been performed (7, 8, 13, 16, 17, 18, 19). To identify directly the sites to which Chls *d* and *f* bind, the structure of a monomeric apo-FRL-PSII complex isolated from a marine, mesophilic cyanobacterium, *Synechococcus* sp. PCC 7335 (hereafter *Synechococcus* 7335), was solved at a global resolution of 2.25 Å using single-particle cryo-EM (18). Compared to a monomer of a typical PSII holocomplex found in a dimeric configuration, the monomeric apo-FRL-PSII complex lacked various transmembrane subunits, the extrinsic subunits (*i.e.*, PsbO, PsbQ, PsbU, and PsbV), and the OEC; however, it contained all four Chl-binding subunits which allowed for the identification of 33 of the 35 Chl sites. By comparing sequences to determine FRL-specific residues, identifying common Chl *d*/*f* chemical environments and employing quantitative map assessments, four Chl *f* molecules and one Chl *d* molecule were assigned, which was consistent with the content of those pigments determined by cofactor analysis (18). The assignments were subsequently corroborated by a larger phylogenetic comparison of FRL-PSII sequences, which also provided insight into their evolution (17). A disadvantage of the apo-FRL-PSII structure was that it lacked PsbH2, the FRL-specific isoform of PsbH. Because all other PSII and photosystem I (PSI) subunits encoded by the FaRLiP gene cluster have associated molecular structures (18, 20, 21, 22, 23), PsbH2 is therefore the last FaRLiP-specific protein to have its structure determined.

To gain insight into the molecular structure of the FRL-PSII holocomplex, we have altered the purification procedure of FRL-PSII from *Synechococcus* 7335 allowing us to isolate a dimeric complex that maintains the PsbH2 subunit, and we solved its cryo-EM structure to a global resolution of 2.6 Å. Whereas PsbH found in VL-absorbing PSII is involved in tuning the energy of the lowest energy Chl *a* molecule found in PSII, PsbH2 lacks this interaction. This suggests that during FaRLiP, the energetic landscape of the antenna Chls is altered to favor energy transfer to Chl *f*. Because identifying Chls *d* and *f* in cryo-EM maps is challenging (24), the new cryo-EM map allows for a reassessment of the Chl type assignments. We performed the strategies previously utilized for this purpose, and observe that two sites in FRL-PSII exhibit CH-O interactions with the formyl moiety of Chl *f*. Because all Chl *d* and Chl *f* molecules exhibit FRL-specific interactions between their formyl moiety and the protein environment, we additionally developed a Python-based tool that can be used to search any set of Chl-containing atomic coordinates for potential H-bond donors to similar formyl moieties. The H-bond search tool is useful for making an initial assessment of structures that bind formylated Chl molecules among the “bulk” Chl *a*. The structural observations are interpreted in the context of other FaRLiP investigations that enhance our understanding of this widespread photobiological acclimation mechanism.

## Results

### Characterization of dimeric FRL-PSII complexes

FRL-PSII was isolated by immobilized metal affinity chromatography from *Synechococcus* 7335-*psbC2*-[His]_10_ cells as described in the Experimental procedures. The room temperature absorbance spectrum shows a FRL absorbance peak maximal at 724 nm, and the 77 K fluorescence emission spectrum (excitation wavelength, 440 nm) shows maximal emission at 739 nm, both characteristic of FRL-PSII complexes characterized previously (Fig. S1) (8, 11, 13, 18). SDS-PAGE showed bands typical for PSII complexes including core subunits, extrinsic subunits, small peripheral transmembrane subunits, and assembly factors (Fig. S2). Mass spectrometry of tryptic peptides was performed which identified various PSII subunits (Table S1). It showed that the FRL-specific subunits PsbA3, PsbB2, PsbC2, PsbD3, and PsbH2 were far more abundant than were their VL-subunit isoforms. It also showed that the PsbO1, PsbV1, and PsbF2 isoforms were more abundant than PsbO2, PsbV2, and PsbF1, respectively, none of which are encoded in a FaRLiP gene cluster. To make an initial assessment of the oligomeric state, the FRL-PSII complexes were negatively stained with uranyl acetate and imaged by transmission electron microscopy (Fig. S3). The images showed relatively monodisperse single particles whose size was similar to those in a sample of dimeric PSII from the mesophilic cyanobacterium *Synechocystis* sp. PCC 6803 (hereafter *Synechocystis* 6803), suggesting that the FRL-PSII sample had maintained a dimeric configuration. Finally, pigment analysis was performed by solvent extraction and subsequent HPLC (Fig. S4). The results were consistent with previously published pigment analyses (8, 11, 13, 18) that suggest the FRL-PSII complexes contain ∼1 Chl *d*, 4 Chl *f*, 30 Chl *a*, and two pheophytin *a* per FRL-PSII complex.

### Structural overview

**Figure 1:**
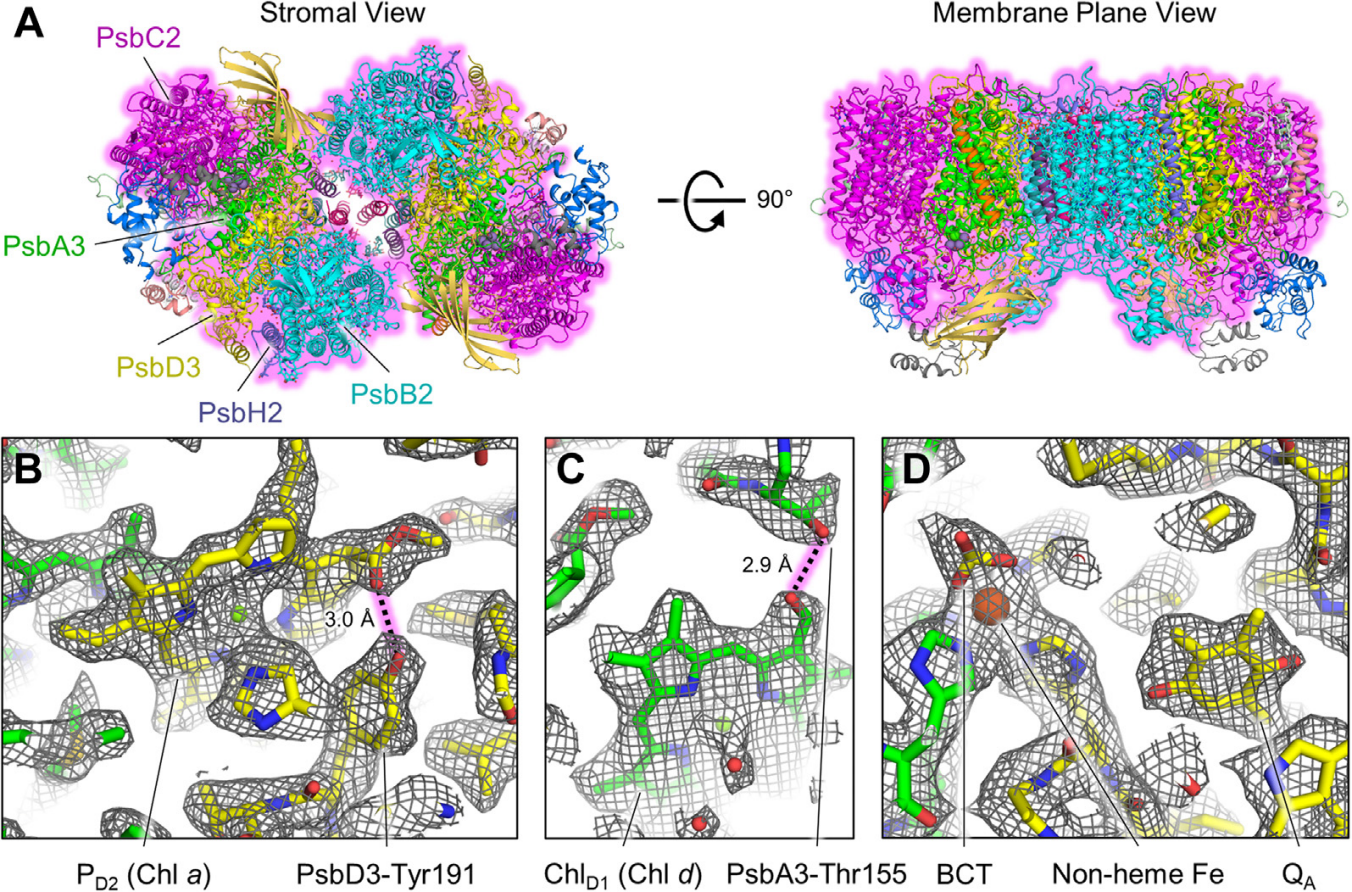
**Cryo-EM structure of dimeric FRL-PSII from *Synechococcus* 7335.***A*, structural model produced from the cryo-EM map. Subunits that are FRL-specific are shown with a *pink glow* and are labeled. Panels (*B*–*D*) show the model within representative regions of the sharpened map. *B*, the FRL-specific H-bond donated from PsbD3-Tyr191 to the 13^2^ methoxycarbonyl moiety of the Chl *a* in the P_D2_ site is shown (13σ). *C*, the FRL-specific H-bond donated from PsbA3-Thr155 to the C2 formyl moiety of the Chl *d* in the Chl_D1_ site is shown (9.5σ). *D*, the vicinity of the non-heme Fe cation, bicarbonate anion (labeled “BCT”), and Q_A_ plastoquinone is shown (11σ). FRL, far-red light; PSII, photosystem II.

The FRL-PSII sample was plunge-frozen in dim green light and imaged for single-particle cryo-EM as described in Experimental procedures (Fig. S5). Micrographs showed monodisperse single particles that after processing yielded a 3D-reconstruction with a global resolution of 2.6 Å with local resolutions spanning the range 2.4 to 4.0 Å (Fig. S6). The map exhibited an obvious dimeric configuration and was therefore processed using C2 symmetry. In each monomer of the FRL-PSII dimer, the following subunits were identified and modeled: PsbA3, PsbB2, PsbC2, PsbD3, PsbE, PsbF2, PsbH2, PsbI, PsbK, PsbL, PsbM, PsbO1, PsbT, PsbU, PsbV1, and PsbX (Fig. S7). These subunits were modeled to coordinate 35 Chl molecules, eight carotenoids, 11 diacyl lipids, two pheophytin *a* molecules, two plastoquinones, two Cl anions, one heme *b*, one heme *c*, one non-heme Fe cation, one bicarbonate anion, one OEC, one *n*-dodecyl β-D-maltoside (β-DM) molecule, and 296 water molecules. The overall structure and three representative map regions are shown in Figure 1. The cryo-EM data and modeling statistics are reported in Table S2. Relative to previously determined structures of dimeric cyanobacterial PSII holocomplexes (25, 26), the structure lacks PsbJ, PsbQ, PsbY, Ycf12, and PsbZ. The extrinsic subunits PsbO1, PsbU, and PsbV1, and the OEC exhibit heterogeneity in their occupancy and positions (Text S1, Figs. S8, S9 and Table S3); therefore, their atomic coordinates should be considered unreliable.

### Assignment of Chl d– and Chl f–binding sites

In the 2.25-Å global resolution structure of apo-FRL-PSII, one site that binds a Chl *d* molecule at high occupancy was assigned in the Chl_D1_ position of the electron transfer chain (18). This is also observed in the dimeric FRL-PSII structure, in which the formyl moiety of Chl *d* accepts an H-bond from the hydroxyl moiety of the sidechain from PsbA3-Thr155 (Fig. 1*C*), which is in turn H-bonded to PsbA3-Tyr120. Importantly, both PsbA3-Thr155 and Tyr120 are FRL-specific and are conserved in the PsbA3 sequences from all cyanobacterial species for which data are available (17). The presence of Chl *d* in the Chl_D1_ site, and additionally the FRL-specific H-bond to P_D2_ (18) which is also conserved in the dimeric FRL-PSII structure (Fig. 1*B*), seem likely to be essential for tuning the redox potentials of the electron transfer chain cofactors so that FRL can drive water oxidation.

Four sites that bind Chl *f* molecules at high occupancy were assigned in the apo-FRL-PSII structure: three in the PsbB2 subunit in sites 605, 608, and 614 and one in the PsbC2 subunit in site 507. To identify possible Chl *f* molecules in the dimeric FRL-PSII map, we performed cone scans as described previously (22) (Fig. S10). In brief, the cryo-EM map around each Chl is scanned at the C7 substituent (always a methyl moiety) and the C2 substituent (a formyl moiety in Chl *f* or a methyl moiety in Chl *a*) at a defined bond geometry and distance (Fig. S11). The C7 cone scan data are used to generate a 3σ null distribution representative of a methyl moiety. When the C2 cone scan data exceed this null distribution, it suggests the presence of a formyl moiety (rather than methyl) and that the site may thus be occupied by Chl *f*.

**Figure 2:**
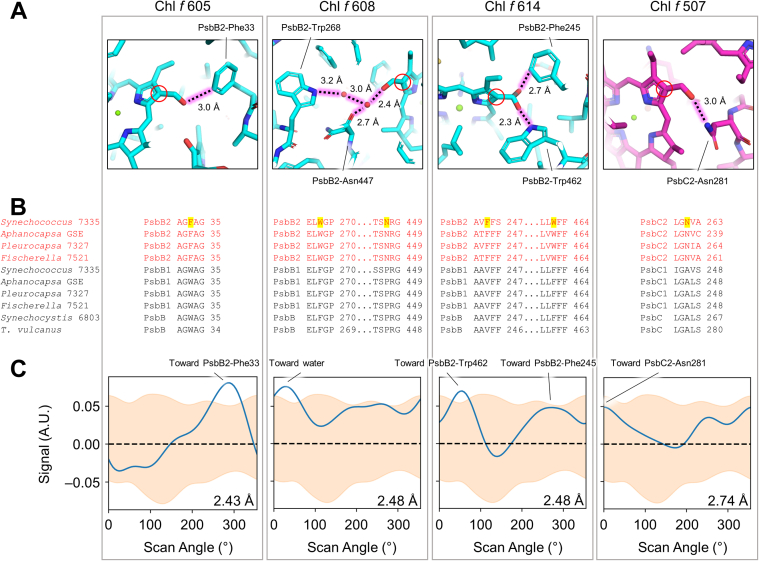
**Chl *f* sites assigned in the dimeric FRL-PSII structure.***A*, H-bonding interactions that confer Chl *f*–binding specificity in the dimeric FRL-PSII structure. FRL-specific residues are labeled and the FRL-specific protein environment interactions are shown with a *pink glow*. For each Chl site, the C2 atom of the tetrapyrrole ring is denoted with a *red circle*. For the panel corresponding to Chl *f* 614, an alternate C2 moiety orientation is shown (*gray*) which corresponds to a minor population with a CH-O interaction from the aromatic ring of PsbC2-Phe245. *B*, sequence conservation of FRL-specific residues that confer Chl *f*–binding affinity. Partial sequence alignments of PsbB and PsbC are shown from four distantly related cyanobacteria that can perform FaRLiP: *Synechococcus* 7335, *Aphanocapsa* sp. GSE-SYN-MK-11-07L (*Aphanocapsa* GSE), *Pleurocapsa* sp. PCC 7327 (*Pleurocapsa* 7327), and *Fischerella thermalis* PCC 7521 (*Fischerella* 7521), and two cyanobacteria that are not able to perform FaRLiP: *Synechocystis* 6803 and *Thermosynechococcus vulcanus* (*T. vulcanus*). FRL-specific sequences are shown in *red font*. Residues that confer Chl *f*–binding specificity are highlighted in *yellow* and correspond to the residues labeled in panel (*A*). *C*, C2 cone scans of the four Chl *f*–binding sites. The methyl distribution is shown in *orange* and the site’s C2 cone scan (formyl if Chl *f*) is shown as a *blue line*. For each scan, the local resolution is shown in the bottom right of the panel. The peak for each scan is labeled with its direction within the protein environment corresponding to panel (*A*). The cryo-EM maps for the four Chl *f* molecules are shown in Fig. S14. FRL, far-red light; PSII, photosystem II.

In PsbB2, all three previously assigned sites, 605, 608, and 614, had C2 cone scan signals greater than the null distribution, and all exhibit FRL-specific interactions that confer Chl *f*–binding specificity (Fig. 2). Near the C2 position of Chl 605, the sidechain of PsbB2-Phe33 is found which in PsbB1 (VL-PSII) is instead a Trp residue. It was previously proposed that the smaller Phe33 allows additional space for the presence of a formyl moiety of the Chl *f* at site 605 (18). The dimeric FRL-PSII structure is consistent with this hypothesis, but we additionally note that the oxygen atom of the formyl moiety is nearly in-plane and within H-bonding distance of the PsbB2-Phe33 aromatic ring. We propose that Chl *f*–binding affinity is conferred by the protein for site 605 by a CH-O interaction *via* the Phe sidechain. Interestingly, another CH-O interaction may also confer some binding affinity for Chl *f*. In the cone scan analysis of site 614, the profile shows two peaks, only one of which is greater than the methyl distribution. This direction corresponds to a formyl moiety positioned to accept an H-bond from the indole nitrogen atom of the FRL-specific residue PsbB2-Trp462. However, the second peak in the cone scan corresponds to a position toward another Phe sidechain, PsbB2-Phe245, which is also FRL-specific. Modeling the formyl moiety in this direction places its oxygen atom in plane and within H-bonding distance of the aromatic ring, consistent with a CH-O interaction. We hypothesize that the formyl moiety of Chl *f* 614 occupies two configurations: a primary one directed toward PsbB2-Trp462 and a secondary one that is lower occupancy directed toward PsbB2-Phe245 (Fig. 2*A*). Thus, it may be that two CH-O interactions participate in Chl *f*-binding in FRL-PSII, in sites 605 and 614.

Another interesting observation is that in structures of FRL-PSI, there was only one Chl *f* molecule that did not exhibit an obvious H-bond, that in site B38 (22, 23). This site was suggested to form a Chl *f* dimer that had been observed spectroscopically with the Chl in site B37 (27). Near the C2 position of B38, a Trp found in VL sequences is instead a Phe found in FRL sequences (Fig. S12). Rather than a CH-O interaction, the orientation of the Phe sidechain is more consistent with a CHO–π interaction between the formyl oxygen atom of the Chl *f* and the conjugated system of the Phe sidechain. Thus, it appears that Phe sidechains may commonly participate in conferring Chl *f*–binding specificity.

Consistent with previous observations, both Chls PsbB2 608 and PsbC2 507 also exhibit FRL-specific H-bonding interactions near their C2 positions (17, 18). PsbC2 507 is the only Chl *f*–binding site for which the peak of its C2 cone scan does not exceed the methyl distribution, although the peak is directed toward the FRL-specific PsbC2-Asn281. As was also the case for the apo-FRL-PSII structure, this site has lower local resolution than the other sites, so it is unsurprising that the C2 formyl moiety is challenging to distinguish from the methyl distribution. Despite this, we believe that the proximity of the FRL-specific residue that provides specificity for Chl *f*-binding at site 507 is strong evidence for its assignment as a Chl *f*–binding site. Additionally, this assignment is supported by recent spectroscopic results showing that the calculated and experimental lifetime for energy transfer from Chl *f* in site 507 to Chl *d* in the Chl_D1_ site of the electron transfer chain are in agreement (19). There were two other sites that exhibited C2 cone scan peaks above the null distribution that are likely to be false-positives due to inaccurate fitting of their tetrapyrrole rings in poorly resolved map regions and/or close proximity to hydrophobic features that do not correspond to FRL-specific alterations, which are shown in Fig. S13. The cryo-EM map regions for the four Chl *f* molecules are shown in Fig. S14.

### An automated H-bond search for assigning Chls b, d, and f

Despite the loss (or absence) of some peripheral subunits, 35 Chl-containing sites were found in each monomer of the dimeric FRL-PSII structure, whereas the apo-FRL-PSII structure contained only 33 (18). The one Chl *d* and four Chl *f* assignments made in the dimeric FRL-PSII structure are consistent with those made in the apo-FRL-PSII structure, and all exhibit FRL-specific interactions from the protein environment with their C2/C3 formyl moiety. This is also the case for the Chl *f*–binding sites in FRL-PSI structures (22, 23) and most Chl *b*–binding sites in structures of light-harvesting complexes. This consistent observation is useful to assist in making similar assignments in other Chl-containing systems. However, because visual inspection of each site is error-prone and subject to bias, we developed an algorithm to automate the search for Chl *b–*, Chl *d–*, or Chl *f–* binding sites (see Experimental procedures and Data S2). The H-bond search identifies nitrogen atoms and oxygen atoms of hydroxyl sidechains within 4.1 Å of the CX^1^ atom of a Chl site (*e.g.*, a search for potential H-bond donors for Chl *f* would use the C2^1^ atom of the Chl). We tested the H-bond search algorithm on two systems for which the Chl type assignments are relatively confident: the structure of Chl *a*/*b*–containing light-harvesting complex II (LHCII, chain A of PDB 2BHW (28)) and the FRL-PSII complex structure reported here (Tables S4 and S5). Of the 14 Chl sites in the LHCII structure, the search for H-bond donors near the C7^1^ atom (positive suggests Chl *b*) correctly assigned 13 (93%) types correctly. Of the 35 Chl sites in the FRL-PSII structure, the search for H-bond donors near the C3^1^ atom (positive suggests Chl *d*) correctly assigned 32 (91%) types correctly, and the search for H-bond donors near the C2^1^ atom (positive suggests Chl *f*) correctly assigned 32 (97%) types correctly. The one Chl *b* incorrectly predicted to be Chl *a* in LHCII has its formyl moiety located toward the bulk solvent (28), so no H-bond donor is located in the deposited coordinates. The three incorrectly predicted Chls in the Chl *d* search of FRL-PSII have potential H-bond donors that are already engaged in H-bonding interactions and are therefore not capable of donating H-bonds to a formyl moiety at that position. The one Chl *f* incorrectly predicted to be Chl *a* in FRL-PSII is Chl 605 whose formyl moiety exhibits only a CH-O interaction to confer specificity for binding as described above. Although some false positives and negatives are observed, the automated system allows for an initial prediction of accuracy >90% for identifying probable sites for the binding of Chls *b*, *d*, or *f*.

### Structure of PsbH2 in the FRL-PSII complex

**Figure 3:**
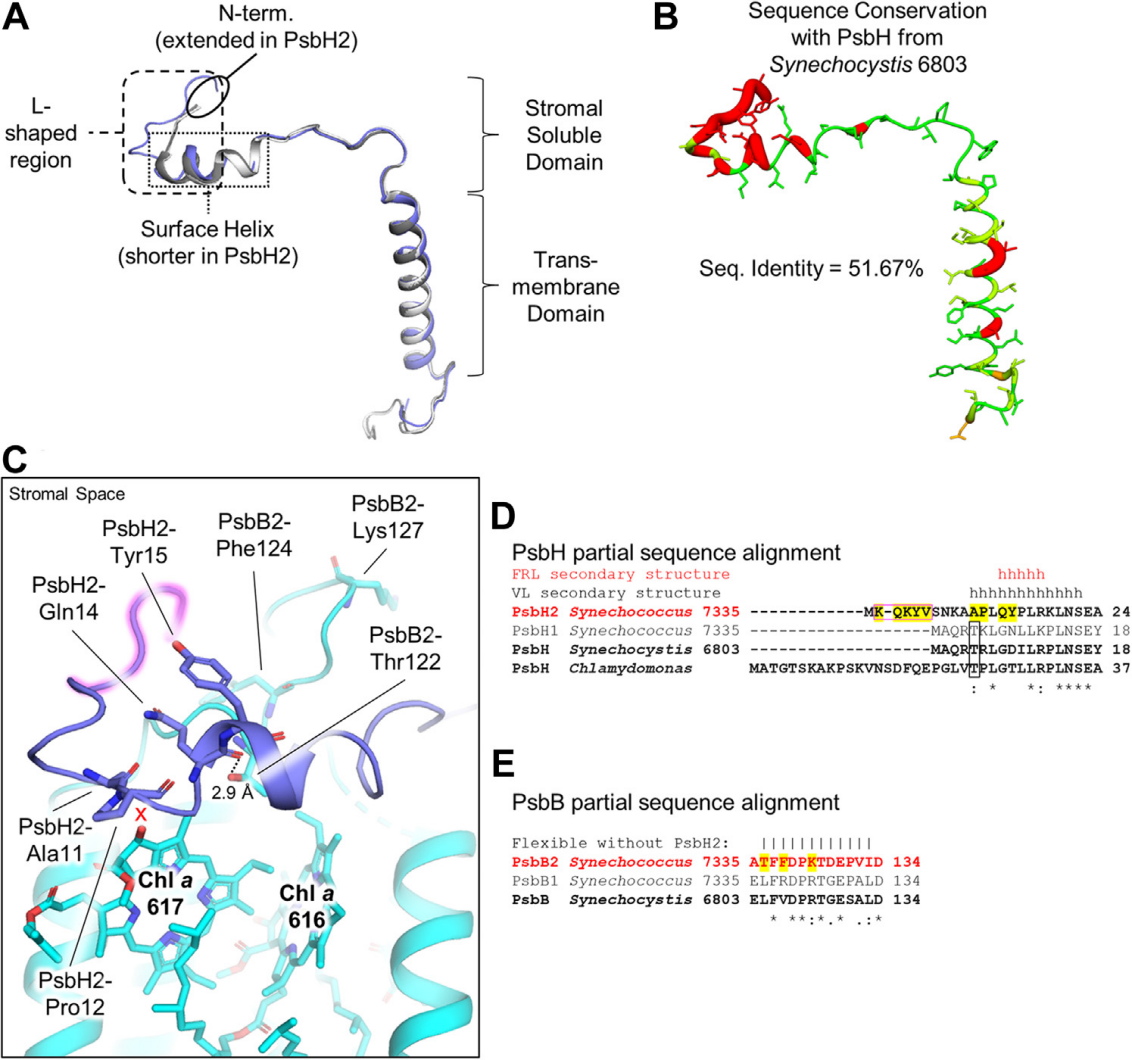
**Structure of PsbH2 in the dimeric FRL-PSII complex and comparison with PsbH from VL-absorbing PSII complexes.***A*, C_α_ superposition of PsbH2 (*blue*) with the PsbH subunits from *Synechocystis* 6803 (*white*) (26) and *Thermosynechococcus vulcanus* (*gray*) (25). Notable structural features of the N-terminal region and the soluble and transmembrane domains are labeled. *B*, structure of PsbH2 colored by sequence conservation with PsbH from *Synechocystis* 6803. Sequence conservation is shown from high (*green*) to none (*red*). *C*, structure of PsbH2 and its interactions with PsbB2. The FRL-specific N-terminal extension is highlighted in *pink*. Other FRL-specific residues are labeled. The *red* “x” corresponds to the region lacking an H-bond to Chl *a* 617 that is observed in non-FaRLiP PSII structures. *D*, partial sequence alignment of PsbH2 with PsbH1 and standard PsbH sequences. Sequences in *bold* have associated structures available. Sequences in *red font* are FRL-specific isoforms. Sequences in *black font* are VL-specific isoforms or non-FaRLiP sequences. Secondary structure is shown above the alignment for FRL and non-FaRLiP cyanobacterial structures, respectively. Whereas the residues composing the transmembrane α-helix (*t*) are aligned, the α-helix on the stromal surface (*h*) near the N-terminus is shorter in FRL sequences. Residues boxed in *pink* correspond to the N-terminal extension found in FRL-specific sequences that is highlighted *pink* in panel (C). Residues highlighted in *yellow* are those conserved in all FRL sequences determined previously (17). The PsbH/PsbH1-Thr sidechain found in non-FaRLiP PSII to H-bond to Chl *a* 617 is boxed. *E*, partial sequence alignment of PsbB2 with PsbB1 and standard PsbB sequences. Sequences in *bold* have associated structures available. Like panel (*D*), sequences in *red font* are FRL-specific isoforms, sequences in *black font* are VL-specific isoforms or non-FaRLiP sequences, and residues highlighted in *yellow* are those conserved in all FRL sequences determined previously (17). Residues unable to be modeled in the apo-FRL-PSII structure that lacked PsbH2 (18) are denoted with a vertical line above the position. These residues were able to be modeled in the dimeric FRL-PSII structure that maintains PsbH2. Sequence alignments were constructed using Clustal Omega (72). FRL, far-red light; FaRLiP, far-red light photoacclimation; PSII, photosystem II.

PsbH is a subunit found in the PSII holocomplexes of all oxygenic phototrophs. It associates with PsbB early in PSII biogenesis, prior even to the association of PsbB with the PsbA and PsbD core subunits (29, 30). In the PSII holocomplex, PsbH is needed to maintain the stability of the bicarbonate anion and therefore influences the Q_A_ to Q_B_ electron transfer rate (31). The FRL-specific isoform of PsbH, called PsbH2, was missing from the apo-FRL-PSII structure reported recently (18), possibly explaining the replacement of the non-heme Fe-bound bicarbonate in that structure with an Asp residue and making PsbH2 the only photosystem subunit encoded by the FaRLiP gene cluster that had not had its structure elucidated previously. In the dimeric FRL-PSII structure, PsbH2 is maintained at high occupancy and bicarbonate is bound to the non-heme Fe (Fig. 1). Like PsbH found in non-FaRLiP structures of PSII, the N-terminal region of PsbH2 contains a soluble domain that stretches across the stromal face of PsbB2, which is followed by the single transmembrane α-helix of the subunit (Fig. 3*A*). The secondary structure of PsbH2 differs from PsbH/PsbH1 of other PSII complexes primarily in its N-terminal region on the cytoplasmic side (Fig. 3*A*). In PsbH/PsbH1 structures from nonacclimated PSII structures, the N-terminal region is L-shaped which is followed by an α-helix that is ∼16 Å long and comprises ∼12 residues. In PsbH2, this L-shape is larger due to a 6-residue N-terminal extension, and the following α-helix is very short, ∼6 Å comprising only ∼5 residues.

To investigate whether these features are conserved in PsbH2 structures from other FaRLiP-capable cyanobacteria or whether they are a consequence of species differences, we constructed sequence alignments of PsbH from a diverse set of oxygenic phototrophs, some capable of and some incapable of performing FaRLiP (Fig. S15). Sequence identity comparisons of PsbH2 with PsbH1 and PsbH of two non-FaRLiP cyanobacteria (Table S6) demonstrate that PsbH2 is more dissimilar to the rest (∼50%) than they are to one another (70–80%). This is consistent with the C_α_ superposition of PsbH2 with the structure of PsbH from either of two non-FaRLiP cyanobacteria (Fig. 3*A*), where the RMSD is ∼0.6 Å, whereas the RMSD of PsbH between the two non-FaRLiP cyanobacteria is very low, only ∼0.2 Å (Table S7). Inspection of the multiple-sequence alignment shows that the N-terminal region is conserved among PsbH2 sequences and is distinct from the N-terminal region conserved in PsbH1 and the PsbH sequences from organisms that do not perform FaRLiP (Fig. S15). Mapping of sequence conservation between PsbH2 and PsbH from *Synechocystis* 6803 allows for visualization of the regions containing major sequence differences (Fig. 3*B*), which correspond to the secondary structural comparison (Fig. 3*A*).

To investigate the functional importance of PsbH2 and to understand how the primary sequence differences give rise to the secondary structural differences compared to other PsbH structures, we inspected the PsbH2 structure in the context of the multiple-sequence alignment (Fig. S15). Residues comprising the N-terminal extension that cause the larger L-shape of the protein found near the N-terminus (Fig. 3*A*) are specific only to PsbH2 sequences, suggesting this feature is conserved in PsbH2 structures of all FaRLiP-capable cyanobacteria. The function of the N-terminal extension is unclear, but it could interact with a FRL-specific phycobiliprotein complex that binds to the stromal surface. Stromal surface residues in PsbB2 and PsbD2 have also been suggested to interact with such a FRL-specific phycobiliprotein complex (17, 18). The location of all FRL-specific stromal surface residues in the FRL-PSII complex is shown in Fig. S16.

Following the N-terminal extension, four FRL-specific residues have been shown to be present in the PsbH2 sequences from all FaRLiP-capable cyanobacteria (17): PsbH2-Ala11, Pro12, Gln14, and Tyr15 (Figs. 3 and S15), suggesting their importance in FRL-PSII. The sidechains of PsbH2-Gln14 and Tyr15 are directed toward the FRL-specific, N-terminal extension, and probably form H-bonding interactions with it, although the specific interactions are unclear due to the lower resolution of the N-terminal extension residues. These, and the rigid PsbH2-Pro12, probably stabilize the L-shaped N-terminal region of PsbH2. In PSII structures from cyanobacteria, algae, and plants that do not perform FarLiP, PsbH2-Ala11 is instead a highly conserved Thr sidechain that donates an H-bond from the hydroxyl oxygen atom of its sidechain to the 13^1^-keto oxygen atom of the Chl *a* molecule in site 617 whose axial ligand is a His sidechain from PsbB (Fig. S17). Notably, H-bonding to this atom has been shown to decrease the Chl site energy (32). The PsbH-Thr sidechain is conserved among PsbH1 sequences (Fig. S15), so this interaction is likely identical for VL-PSII complexes of cyanobacteria capable of performing FaRLiP. Due to the presence of PsbH2-Ala11 rather than Thr, no H-bonding interaction is observed from PsbH2 to Chl *a* 617 (red “x” in Fig. 3*C*), as had been suggested previously by homology modeling (13, 17). Furthermore, the stabilization conferred by PsbH2-Pro12, Gln14, and Tyr15 probably helps to block waters from accessing this region that could donate an H-bond to the 13^1^-keto oxygen atom of Chl *a* 617. Thus, the conserved Ala11, Pro12, Gln14, and Tyr15 residues of PsbH2 in FRL-PSII appear to confer a higher site energy for Chl *a* 617.

In the structure of apo-FRL-PSII in which PsbH2 was missing, a looping region of PsbB2 between its second and third transmembrane helices could not be modeled (18), presumably due to its flexibility (labeled as vertical lines in Fig. 3*E*). This region could be modeled in the dimeric FRL-PSII structure in which PsbH2 is bound, demonstrating that PsbH2 stabilizes the PsbB2 loop. This looping region contains three residues that are also FRL-specific: PsbB2-Thr122, Phe124, and Lys127 (labeled in Fig. 3*C* and highlighted in Fig. 3*E*). The hydroxyl moiety of the PsbB2-Thr122 sidechain donates an H-bond to the backbone carbonyl oxygen atom of PsbH2-Gln14, which is also FRL-specific. PsbB2-Phe124 appears to be involved in stabilizing the extended N-terminal region of PsbH2. PsbB2-Lys127, whose sidechain is directed out into the stromal space, may be involved in binding the FRL-phycobiliprotein complex as we suggested for the N-terminal extension of PsbH2 above.

## Discussion

**Figure 4:**
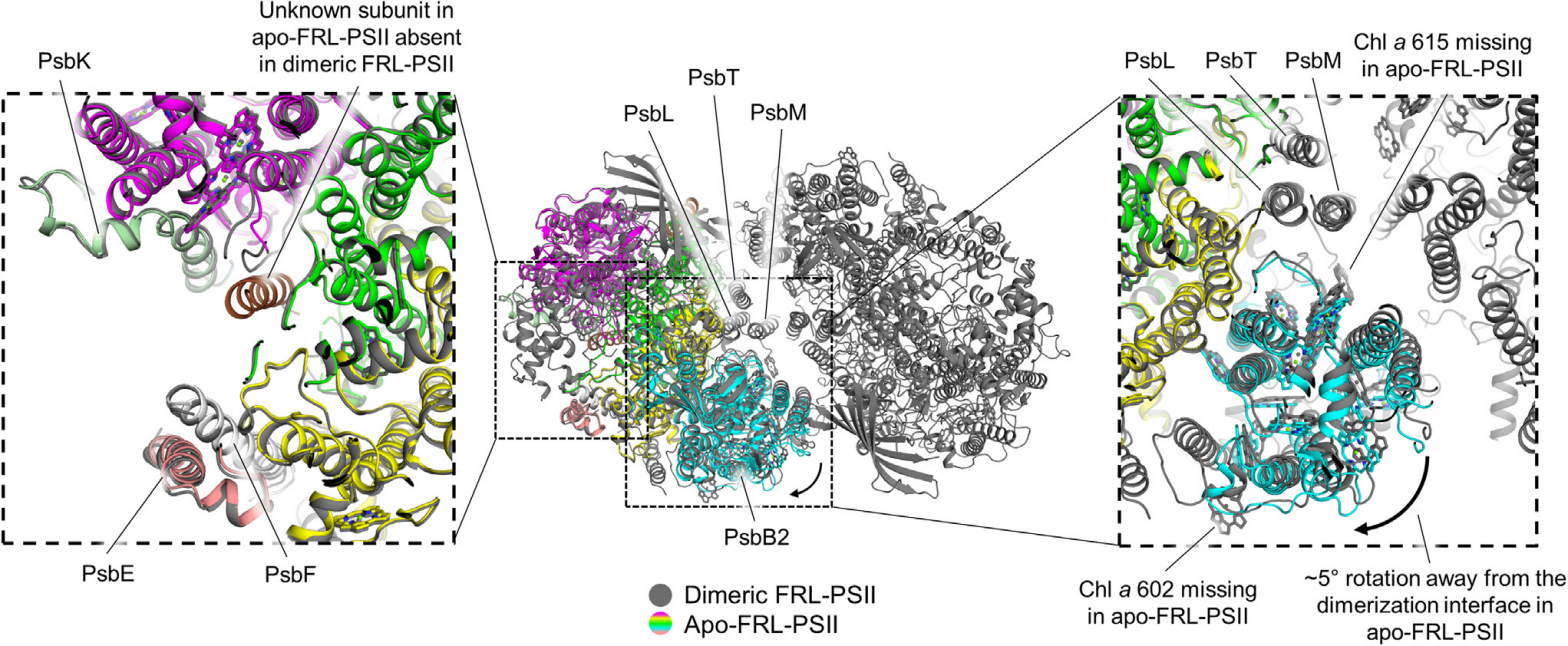
**Apo-FRL-PSII structure comparison with the dimeric FRL-PSII structure.** The apo-FRL-PSII structure (colored) is superimposed on the PsbA3 and PsbD3 subunits of the dimeric FRL-PSII structure (*gray*). The *left inset* shows the region of the unidentified single transmembrane α-helix observed in the apo-FRL-PSII structure (*brown*). This is not present in the dimeric FRL-PSII structure despite a similar subunit composition in that region between the two structures. In the dimeric FRL-PSII structure, two lipids occupy the space of the unknown subunit which is shown in Fig. S17. The right inset shows the region of PsbB2. In the absence of the dimeric state and PsbL, PsbM, and PsbT subunits, the PsbB2 subunit (*cyan*) is rotated away from the would-be dimerization interface ∼5°. Additionally, two Chl *a* molecules are present in the dimeric FRL-PSII structure but absent in the apo-FRL-PSII structure. FRL, far-red light; PSII, photosystem II.

The apo-FRL-PSII complex described previously (18) contained subunits PsbA3, PsbB2, PsbC2, PsbD3, PsbI, PsbK, and an unidentified single transmembrane α-helix subunit (Fig. 4). The dimeric FRL-PSII structure described here is more complete than the apo-FRL-PSII structure; in addition to the dimeric configuration, each monomer comprises PsbA3, PsbB2, PsbC2, PsbD3, PsbE, PsbF2, PsbH2, PsbI, PsbK, PsbL, PsbM, PsbO1, PsbT, PsbU, PsbV1, and PsbX. Furthermore, the apo-FRL-PSII structure maintained 33 of the 35 Chl sites, whereas the dimeric FRL-PSII structure maintains all 35 Chl sites (Fig. 4). Interestingly, the unidentified single transmembrane α-helix subunit modeled as a poly-Ala α-helix in the apo-FRL-PSII structure (18) is not present in the dimeric FRL-PSII structure. This is somewhat surprising because the subunit composition of the environment near where the single-transmembrane α-helix was modeled in the apo-FRL-PSII structure is identical to that of the dimeric FRL-PSII structure (Fig. 4, left inset). However, in the dimeric FRL-PSII structure, the site of the unknown subunit is occupied by two lipid molecules, a sulfoquinovosyl-diacylglycerol and a digalactosyl-diacylglycerol (DGD), one on each side of the complex (Fig. S18). The DGD appears analogous to a DGD identified in the apo-FRL-PSII structure that is shifted into the site where the single transmembrane α-helix was modeled. The sulfoquinovosyl-diacylglycerol does not have an analogous lipid in the apo-FRL-PSII structure but is conserved with a lipid found in other dimeric PSII structures such as those from *Synechocystis* 6803 (26) and *Thermosynechococcus vulcanus* (25). The identity of the single transmembrane α-helix subunit observed in the apo-FRL-PSII structure thus remains unknown. However, we suggest that perturbation of native lipids and extensive subunit loss might have resulted in the promiscuous, nonphysiological binding of one or more single transmembrane α-helix subunits at that site at low occupancy. Future studies that preserve the native lipid environments may help to clarify this issue.

By superimposing the central PsbA3 and PsbD3 subunits of the apo-FRL-PSII structure with those from a monomer of the dimeric FRL-PSII structure, it is also observed that the PsbB2 subunit is rotated ∼5° toward the dimerization interface when the complex is in a dimeric configuration (Fig. 4, right inset). This rotation in the apo-FRL-PSII structure is probably due to the absence of stabilizing interactions. The apo-FRL-PSII structure not only lacked the other monomer of the dimer that would interact with PsbB2 but also the subunits PsbM, PsbL, and PsbT, toward which the PsbB2 subunit is rotated in the apo-FRL-PSII structure (Fig. 4). PsbM, PsbL, and PsbT are all small subunits with single transmembrane α-helices that stabilize the dimerization interface, so these interactions underscore the importance of PSII dimerization in stabilizing at least PsbB2 in FRL-PSII.

It is noteworthy that two main alterations were made to the protein isolation procedure to produce dimeric FRL-PSII to retain the dimeric configuration, thus avoiding the monomeric oligomeric state reported previously (18). First, the isolation buffers contained glycine betaine, which is known to be a potent stabilizer of PSII (33). Second, the apo-FRL-PSII was isolated first using immobilized metal affinity chromatography and was further purified by sucrose density gradient centrifugation. The absence of stabilizing glycine betaine and the sucrose gradient centrifugation step probably caused subunit loss when the apo-FRL-PSII was isolated, but it seems to have resulted in greater homogeneity, which is reflected in the higher resolution that was achieved, 2.25 Å. For the dimeric FRL-PSII isolation, glycine betaine was included throughout the protocol and the sucrose gradient step was omitted. Although the isolated sample is dimeric and contains more subunits per monomer, the occupancy of some subunits is quite variable, suggesting that the ensemble single particle data used to produce the cryo-EM map is heterogeneous as is exemplified by the lower resolution achieved, 2.6 Å. These observations highlight the sensitivity of PSII preparations. As described more extensively in Text S1, we can suggest several factors that contribute to the disassembly of FRL-PSII, which include the following: (a) the protein complex being isolated from a mesophilic cyanobacterium, whereas complexes from a thermophilic cyanobacterium might be more robust, (b) differences in subunit affinity among species that are not based on thermostability, and/or (c) differences in subunit stability between VL- and FRL-PSII subunits, given that many subunits are common to both types of PSII.

The Chl *d* and Chl *f* assignments in the dimeric FRL-PSII structure are consistent both with those assigned previously in the apo-FRL-PSII structure (18) and with extensive phylogenetic analyses of FRL-specific residues (17). These Chl *d*– and Chl *f*–binding sites always exhibit FRL-specific features of the protein environment that interact with the formyl moiety of the C3 or C2 positions of Chl *d* and Chl *f*. Similar features are also observed in the majority of cases in LHCI/II structures for Chl *b*, which has a formyl moiety at the C7 position of the tetrapyrrole ring. These observations demonstrate that, at least in systems for which Chl *a* is the “bulk” pigment, some proteins have evolved to confer binding specificity for the less abundant Chls *b*, *d*, or *f* by positioning H-bond donors or other interacting residues (*e.g.*, the CH-O interaction from a Phe sidechain to the formyl moiety of Chl *f* 507) near the corresponding sites. This is advantageous, because there are probably some sites that require the presence of a specific Chl type for the system to function optimally or properly. For example, we hypothesize that water oxidation would be inefficient without the presence of Chl *d*-binding in the Chl_D1_ site of FRL-PSII where specificity for Chl *d* is conferred by H-bond donation to the C3 formyl moiety from a Thr residue in PsbA3 (Fig. 1). We have leveraged this design principle, the frequent positioning of H-bond donors to confer Chl type specificity, to create a useful H-bond search algorithm that predicts the Chl type bound in the sites of two systems, LHCII and the dimeric FRL-PSII complex, that assigned Chl types with >90% accuracy. This tool can easily be used to identify H-bond donors in the structures of other similar systems in the future.

The arrangement of the FRL-specific residues on the stromal surface of FRL-PSII is interesting because they most likely interact with FRL-specific residues of the modified phycobiliprotein complex that is specifically produced in FRL (34, 35, 36). The FRL-specific phycobiliprotein complex in *Synechococcus* 7335 is probably composed of bicylindrical allophycocyanin cores (35) configured similarly to the allophycocyanin cores found in larger phycobilisomes. We measured the approximate dimensions of the two cylindrical cores of the phycobilisome structure published from *Synechococcus* sp. PCC 7002 that interact with the stromal surface and the bases of those cylindrical cores that are probably within a reasonable distance to interact with the stromal side of FRL-PSII. Only one orientation of a bicylindrical core seems capable of interacting with most of the FRL-specific residues on the stromal surface of FRL-PSII (Fig. S16). Interestingly, this orientation does not simply align one monomer of FRL-PSII with one cylinder but rather exhibits a crossed pattern in which both cylinders partially interact with both of the FRL-PSII monomers. This orientation is similar to that observed by atomic-force microscopy of thylakoid membrane surfaces of FaRLiP strains (11), by computational modeling (37, 38), and from a subtomogram average of cyanobacterial phycobilisome-PSII complexes using cryo-electron tomography (39), although those data were very low resolution. Higher resolution structural data of phycobiliproteins complexed with PSII are required for a better understanding of the binding orientations of these protein complexes and the energy transfer pathways between them.

The observation that the site energy of Chl *a* 617 is likely increased during FaRLiP and that there are FRL-specific residues on the stromal surface are especially significant in the context of energy transfer from a FRL-absorbing phycobiliprotein complex. The presence of FRL-specific residues on the stromal surface suggests that at least part of the phycobiliprotein complex binds in the vicinity of Chl *a* 617 (Fig. S16). Strong evidence supports Chl *a* 617 as being the lowest energy Chl site in typical Chl *a*–containing PSII complexes from various species (40, 41, 42, 43, 44), so it may be that when cells are grown in VL, energy enters PSII from the phycobilisome *via* Chl *a* 617. In FRL-PSII, the site energy of Chl *a* 617 is increased, but the energy absorbed by the FRL-phycobiliprotein complex is lower, so the energy transfer pathway must be different in the FRL-absorbing system. Consistent with this hypothesis, Mascoli *et al*. recently showed that energy absorbed by the FRL-phycobiliprotein complex is trapped in FRL-PSII very quickly, faster than the analogous process that occurs under VL conditions, and that energy transfer likely occurs *via* the Chl *f* bound by the PsbC2 subunit that is well connected to the electron transfer chain (19). Thus, we can expand upon this observation by suggesting that the increase in site energy of Chl *a* 617 results in the redirection of energy flow into the FRL-PSII core. Energy is directed instead to Chl *f* 507 bound by PsbC2 and subsequently to the Chl *d* in the Chl_D1_ site of the electron transfer chain which initiates charge separation as we suggested previously (18). Importantly, the residues that are involved in the binding of the Chl *f* in PsbC2 are absolutely conserved in all available PsbC2 sequences and were calculated to have been present in an ancestral precursor to extant PsbC2 subunits (17). Due to the importance of this site in energy transfer from the FRL-phycobiliprotein complex, it suggests that the FRL-phycobiliprotein complex evolved first and that energy transfer to the core was made more efficient by the evolution of Chl *f* binding in site 507 of PsbB2. This would have occurred after the evolution of Chl *d* binding in the electron transfer chain that allows for water oxidation (17).

The spectral deconvolution of the low temperature absorption spectrum of FRL-PSII performed by Nürnberg *et al*. suggested that a single Chl *d* or Chl *f* molecule is involved in electron transfer, a single Chl *d* or Chl *f* molecule is involved in bridging energy transfer from the phycobiliprotein complex to the electron transfer chain, and three Chl *d* or Chl *f* molecules serve as the lowest energy light-harvesting pigments in the complex (13). As described above, the evidence is strong for assigning the Chl *d* in the Chl_D1_ site as the one involved in electron transfer and for assigning the Chl *f* bound by PsbC2 as the one involved in energy transfer from the phycobiliprotein complex. By process of elimination, this leaves the three Chl *f* molecules bound in the PsbB2 subunit as those which serve as the lowest energy light-harvesting pigments in the complex. This is also consistent with the observations by Mascoli *et al*., who also showed that the Chl *f* molecules bound by PsbB2 are not involved in bridging energy transfer from the phycobiliprotein complex to the electron transfer chain and that they have a much slower trapping time than energy arriving from the Chl *f* in PsbC2 (19). PsbB2 was suggested to have evolved later in the evolution of the FRL-specific subunits in FRL-PSII, and some residues involved in conferring binding specificity for Chl *f* exhibit more variability in their conservation (17), so these most bathochromically shifted chromophores probably evolved more slowly. The slow evolution of the Chl *f* molecules bound by PsbB2 makes sense; on one hand, they access the lowest energy light that can be used to drive photochemistry, but on the other hand, the trapping efficiency from this population is very low. In other words, nature has sacrificed the trapping efficiency of this light-harvesting system to enhance its absorption cross section. This nicely exemplifies the diversity of light-harvesting strategies in nature, and the fine balancing of resource availability, environmental plasticity, and efficiency that can be accomplished over time by evolution.

## Experimental procedures

### Strain and growth conditions

Cells of *Synechococcus* 7335 *psbC2*-His/Km (hereafter S7335 *psbC2*-His) were grown at room temperature (∼25 °C) in ASNIII medium amended with kanamycin (50 μg mL^–1^) and were sparged with 1% (v/v) CO_2_ in air, as described previously (18). The starting liquid culture of S7335 *psbC2*-His strain was grown in white light. For growth in FRL, liquid cultures that were first adapted to red light were diluted to about OD_750_ = 0.2 to initiate the FRL cultures (18, 45). The large liquid cultures of strain S7335 *psbC2*-His were grown with sparging by 1% CO_2_ in air and continuous stirring in FRL, which was provided by the LED panels with emission centered at 720 nm and/or by filtering halogen light with a combination of green and red plastic filters to provide FRL at ∼20 to 28 μmol photons m^–2^ s^–1^ (46). To obtain complete acclimation to FRL, cells were diluted, and the medium was refreshed at 2-week intervals, and grown continuously then in FRL. FRL-cells of *Synechococcus* 7335 *psbC2*-His were harvested from liquid cultures grown in FRL for 10 weeks or more. Cells were washed once in the MES buffer (50 mM 2-(*N*-morpholino) ethanesulfonic acid (MES)), pH = 6.5, 1.2 M glycine betaine, 15 mM CaCl_2_, 10 mM MgCl_2_ prior to storage at −80 °C.

### Purification of FRL-PSII complexes

For isolation of FRL-PSII complexes, cells grown in FRL were resuspended in cell lysis buffer, which was 50 mM MES/pH = 6.5, 1.2 M glycine betaine, 15 mM CaCl_2_, 10 mM MgCl_2,_ 10% glycerol, and 0.1 mM PMSF. Cells were lysed by four passages through a chilled French pressure cell operated at 138 MPa. Unbroken cells and large cell debris were removed by centrifugation (4284*g*). Total membranes were then pelleted by ultracentrifugation at 126,000*g* for 1 h. Membranes were resuspended in the photosystem purification buffer (PPB), which was 50 mM MES/pH = 6.5, 1.2 M glycine betaine, 15 mM CaCl_2_ and 10 mM MgCl_2_, 10% glycerol, and solubilized at 4 °C in dark for 1 h by addition of β-DM to a final concentration of 0.6% (w/v). After removal of insoluble debris by centrifugation (10,967*g*), the solubilized membranes were diluted two-fold (v/v) with PPB buffer containing 1 mM L-histidine and then loaded onto a Ni-resin column pre-equilibrated with the PPB buffer. The column was washed with 8-column volumes of the PPB buffer supplemented with 2 mM L-histidine and 0.03% (w/v) *β*-DM. FRL-PSII complexes were eluted with the PPB buffer supplemented with 50 mM L-histidine and 0.03% (w/v) *β*-DM. The eluate containing FRL-PSII was dialyzed against prechilled PPB buffer containing 0.03% β-DM and concentrated using Millipore centrifugal filter units. The purified FRL-PSII isolation evolved oxygen at ∼500 μmol O_2_ (mg Chl)^–1^ h^–1^ as measured with a Clark-type electrode.

### Spectroscopic and analytical measurements

Absorption spectroscopy and fluorescence emission spectroscopy at low-temperature (77 K) were measured for the purified FRL-PSII complexes (Fig. S1), as described previously (46). Oxygen evolution activity of the purified FRL-PSII was measured using a Hansatech Oxygen Electrode System (Hansatech). Pigment extraction and reversed-phase, HPLC analysis of the FRL-PSII complexes were performed (Fig. S4), as described previously (46, 47). The purity of the purified FRL-PSII complexes was resolved by SDS-PAGE (Fig. S2) (48), and the protein composition of the purified FRL-PSII sample was detected through tryptic peptide fingerprinting by mass spectrometry (Table S1), as described previously (18, 46).

### Cryo-EM grid preparation

The FRL-PSII sample was concentrated to ∼0.65 mg Chl mL^–1^ using a 100,000 Da MWCO (Millipore) centrifugal filter. A holey-carbon Quantifoil 2/1 Au 300-mesh grid (Electron Microscopy Sciences) was glow-discharged for 60 s at 25 mA using a PELCO easiGlow (Ted Pella) and inserted into a Vitrobot Mark IV system (Thermo Fisher Scientific). The FRL-PSII sample (3 μL at ∼0.65 mg Chl mL^–1^) was applied to the grid under dim green light with the Vitrobot set to 4 °C and 100% humidity. The grid was blotted for 3 s, plunged into liquid ethane, and transferred to liquid nitrogen until imaging.

### Cryo-EM data collection

The FRL-PSII sample was imaged with an FEI Titan Krios transmission electron microscope operated at 300 kV with a Gatan K3 direct electron detector. The nominal magnification was set to 105,000 × with a super-resolution pixel size of 0.416 Å, the defocus range was set to −1.2 to −2.2 μm, and the GIF slit size was 20 eV. Data were collected using a dose rate of 23.7 e^−^ physical pixel^−1^ s^−1^. The total exposure time was 1.2 s per exposure and the total dose was 41.1 e^−^ (Å) ^−2^. Eleven thousand four hundred forty-two micrograph movies (50 images per stack) were collected using SerialEM.

### Single particle cryo-EM data processing

Cryo-EM data were processed using RELION 3.1 (49). An example micrograph image, representative 2D classes, and the workflow of data processing are shown in Fig. S5. Movie frame alignment, correction, and dose-weighting were performed using MotionCor2 (50). Ctffind-4.1.13 (51) was used to estimate the contrast transfer function. Approximately, 800 particles were selected manually and their 2D classes were used as templates for Autopicking. The initial Autopicking job selected 2,467,501 coordinates. Two rounds of 2D classification yielded 844,312 particle coordinates. An *ab initio* initial model was created from this particle set and two rounds of subsequent 3D classification yielded 90,191 particles that reconstructed to a global resolution of 3.94 Å. Iterative contrast transfer function refinement and Bayesian Polishing led to a map at a global resolution of 2.71 Å. Detergent micelle subtraction and final refinement resulted in a map at a global resolution of 2.62 Å based on the Gold standard Fourier Shell Correlation cutoff criterion (49,52).

### Structure modeling

An initial monomeric model was generated by using the subunits from the apo-FRL-PSII structure, which contained PsbA3, PsbB2, PsbC2, PsbD3, PsbE, PsbF1, PsbI, and PsbK and homology models of PsbH2, PsbL, PsbM, PsbO1, PsbT, PsbU, PsbV1, and PsbX created using SwissModel (53) using the analogous subunits of the PSII holocomplex from *Synechocystis* 6803 (26) as templates. The homology model was fit into the map using UCSF Chimera (54) and manually edited using Coot version 0.8.6.1 (55). Well-resolved regions of the structure were manually edited by visualizing the structure within the sharpened map, and poorly resolved regions (*e.g.*, the extrinsic subunits PsbO1, PsbU, and PsbV1) were manually edited by visualizing the structure within the unsharpened map. Due to the low occupancy of the extrinsic subunits, some sidechains of the residues were trimmed, and some regions were not modeled (Fig. S9), especially in the PsbO1 subunit whose secondary structure is a β-barrel which is known to be more challenging to model at intermediate resolutions compared to α-helices (56). For modeling of the OEC, metal ions were manually placed using Coot (55) and fit into their corresponding map region using the “fit in map” function of UCSF Chimera (54). Phenix *real_space_refine* was used for automated refinement of the structure (57). The final refinement and modeling statistics of which are in Table S2.

### Cone scans

Cone scans were performed as described previously (22). The cryo-EM map for each Chl molecule was aligned to a reference Chl according to its fitted coordinates and inverted using the program suits CCP4 (58), Rave (59), and Phenix (60). After scaling, the map data were extracted every 5° at the bond geometry of a formyl moiety at the C2^1^ and C7^1^ positions (Fig. S11). The C7 scan data were used to generate a null distribution modeled by the equation *μ* + 3*σ*, where *μ* is the distribution mean and *σ* is the distribution SD. The C2 scan data were compared against the null distribution (Fig. S10). These data and analysis are provided as a Jupyter Notebook in Data S1.

## Data availability

The cryo-EM structure of dimeric FRL-PSII has been deposited in the Protein Data Bank with accession code 8EQM and its corresponding map in the Electron Microscopy Data Bank with accession code EMD-28539.

## Supporting information

This article contains supporting information. References (61, 62, 63, 64, 65, 66, 67, 68, 69, 70, 71) are cited in the Supporting Information.

## Conflict of interest

The authors declare that they have no conflicts of interest with the contents of this article.

## Supporting information

Fig. S5

Supplementray data 1
