# Supplementary material for: Structure of a dimeric photosystem II complex from a cyanobacterium acclimated to far-red light
Source: J Biol Chem. 2022 Dec 20;299(1):102815. doi: 10.1016/j.jbc.2022.102815 (PMC9843442; doi:10.1016/j.jbc.2022.102815)
Supplement: Fig. S5 [file mmc1.doc]

**Supporting Information for**

Structure of a dimeric photosystem II complex from a cyanobacterium acclimated to far-red light

Christopher J. Gisriel^1^,*, Gaozhong Shen^2^, David A. Flesher^3^, Vasily Kurashov^2^, John H. Golbeck^2,4^, Gary W. Brudvig^1,3^, Muhamed Amin^5,6,7^, and Donald A. Bryant^2^,*

**Affiliations:**

^1^Department of Chemistry, Yale University, New Haven, CT 06520, USA.

^2^Department of Biochemistry and Molecular Biology, The Pennsylvania State University, University Park, PA 16802, USA.

^3^Department of Molecular Biophysics and Biochemistry, Yale University, New Haven, CT 06520, USA.

^4^Department of Chemistry, The Pennsylvania State University, University Park, PA 16802, USA.

^5^Department of Sciences, University College Groningen, University of Groningen, Hoendiepskade 23/24, 9718 BG, Groningen, NLD.

^6^Rijksuniversiteit Groningen Biomolecular Sciences and Biotechnology Institute, University of Groningen, Groningen, NLD.

^7^Center for Free-Electron Laser Science, Deutsches Elektronen-Synchrotron DESY, Notkestrasse 85, 22607, Hamburg, GE.

*To whom correspondence should be addressed: C.J.G., [christopher.gisriel@yale.edu](mailto:christopher.gisriel@yale.edu) and D.A.B., [dab14@psu.edu](mailto:dab14@psu.edu)

**Supplemental Text S1, Supplemental Figures S1 to S17, Supplemental Tables S1 to S7, and Supplementary Data S1 to S2**

**Text S1. Subunit loss and heterogeneity.**

**Figure S1. Absorption and fluorescence spectra of dimeric FRL-PSII complex from *Synechococcus* 7335.**

**Figure S2. SDS-PAGE analysis of the purified FRL-PSII complexes from *Synechococcus* 7335.**

**Figure S3. Transmission electron microscopy of negatively-stained FRL-PSII complexes from *Synechococcus* 7335 and comparison with dimeric PSII complexes from *Synechocystis* 6803.**

**Figure S4. Pigment analysis of the purified FRL-PSII complexes from *Synechococcus* 7335.**

**Figure S5. Cryo-EM data and processing workflow of the dimeric FRL-PSII structure.**

**Figure S6. Resolution of the FRL-PSII cryo-EM map.**

**Figure S7. Subunits modeled in the cryo-EM map.**

**Figure S8. Consequences of low occupancy extrinsic subunits.**

**Figure S9. Modeled residues in the extrinsic subunits.**

**Figure S10. Cone scans of Chl sites.**

**Figure S11. Cone scan measurement diagram.**

**Figure S12. OH–π interaction in position B38 of FRL-PSI.**

**Figure S13. False positive cone scan sites.**

**Figure S14. Cryo-EM map regions for the Chl *f* assignments.**

**Figure S15. Multiple sequence alignment of PsbH and partial sequence alignment of PsbB.**

**Figure S16. FRL-specific PSII residues on the stromal side and suggested region of FRL-phycobiliprotein complex binding to FRL-PSII.**

**Figure S17. Structural conservation of PsbH-Thr H-bonding with Chl *a* 617.**

**Figure S18. Lipids occupying the region of the dimeric FRL-PSII structure in the site where the unknown subunit was present in the apo-FRL-PSII structure.**

**Table S1. Major subunits identified by tryptic peptide fingerprinting and MS/MS mass spectrometry of the dimeric FRL-PSII isolation.**

**Table S2.** **Cryo-EM data statistics for the dimeric FRL-PSII complex.**

**Table S3.** **Metal-metal distances in the OEC of PSII structures.**

**Table S4. Automated H-bond search for Chl *b* in LHCII.**

**Table S5. Automated H-bond search for Chls *d* and *f* in the dimeric FRL-PSII structure.**

**Table S6. Sequence identity of PsbH2 with PsbH from two representative non-FaRLiP cyanobacteria.**

**Table S7. C_α_ superposition RMSD of PsbH2 with PsbH from two representative non-FaRLiP cyanobacteria.**

**Supplementary Data 1. Jupyter Notebook for the cone scan analysis and cone scan data files (external file).**

**Supplementary Data 2. GitHub link for H-bond search.**

**Supporting Text**

**Text S1. Subunit loss and heterogeneity.**

The three extrinsic subunits, PsbO1, PsbU, and PsbV1, were present in the cryo-EM map but appeared to be very low occupancy (i.e., their presence is variable in the particles used for cryo-EM). Based on the intensity of the map regions, their occupancy is estimated to be ~25%. Correspondingly, their local resolution is near the low end of the local resolution range in the map (**Fig. S6B**). Probably due to the heterogeneity of these extrinsic subunits, the OEC and coordinating water molecules are also poorly resolved. Although the cations composing the OEC could be modeled (see **Experimental Procedures**), the bridging oxo atoms could not be visualized and were therefore omitted. The metal cations of the OEC were modeled using the peak centroids of their corresponding map regions (see **Experimental Procedures**), but those differ substantially from the corresponding coordinates in PSII holocomplex structures (**Table S3**), and probably represent a superposition of different active site configurations/compositions. The residue PsbA-His332, which ligates the OEC in PSII holocomplex structures (25), has previously been observed in a rotated orientation nearby Cl^–^1 when the OEC is absent (62, 63). In the dimeric FRL-PSII structure, this residue is best modeled in an intermediate position between those two positions (**Fig. S8A**), also reflecting the heterogeneity of the OEC in the dimeric FRL-PSII structure. Additionally, the two monomers of the dimer are tilted relative to known PSII holocomplex structures about 5°, increasing the distance between the lumenal domains of the two monomers (**Fig. S8B**). Based on these observations, we hypothesize that extrinsic subunit loss during purification and/or cryo-EM preparation destabilized the dimerization interface and caused heterogeneity in the OEC. Consistent with this hypothesis, the PsbO1 subunit especially is known to be important for OEC stability (64) and has been suggested to be involved in stabilizing the dimeric configuration (62, 65, 66).

The cause of the subunit loss described is unclear. It is known that thermostable proteins often exhibit more stable hydrophobic interactions relative to analogous proteins from mesophilic organisms (67–69). Thus, as a mesophilic cyanobacterium, protein complexes from *Synechococcus* 7335 may not be as robust as those found in thermophilic cyanobacteria such as *Thermosynechococcus vulcanus* or *Thermosynechococcus elongatus*. An observation that supports this hypothesis is that recent cryo-EM structures of FRL-PSI from mesophilic *Leptolyngbya*-like spp. (growth temperature = ~25 °C) exhibited very low occupancy of the PsaF2 and PsaJ2 subunits (21, 23), yet the FRL-PSI structure produced from a thermophilic *Fischerella* sp. (growth temperature up to 55 °C) maintained very high occupancy of those subunits (32), despite the purification procedures being very similar. On the other hand, the purification procedure used to isolate the dimeric FRL-PSII complex from *Synechococcus* 7335 was similar to that used to isolate PSII holocomplexes from *Synechocystis* 6803 that recently led to solving its cryo-EM structure to high resolution with high occupancy of the extrinsic subunits and OEC (26). This suggests against the possibility that dimeric FRL-PSII loses subunits more easily than thermophilic cyanobacteria due to a lack of thermostability. Another possibility or contributing factor could be that there are variable subunit binding affinities for PSII subunits among different cyanobacteria. This is supported by the observation that the PsbQ subunit binds with higher affinity in *Synechocystis* 6803 than it does in thermophilic cyanobacteria (70). A third possible cause of subunit loss in the dimeric FRL-PSII complex is that it could be less stable than the PSII complex found when the *Synechococcus* 7335 cells are grown in VL; about 80% of the FRL-PSII complex is composed of FRL-specific subunits, so it could easily be envisioned that the VL-PSII and FRL-PSII complexes differ in their stability. Future experiments that determine PSII stability among organisms and/or differential expression conditions may help to elucidate the likelihood of these possibilities.

**Supporting Figures**


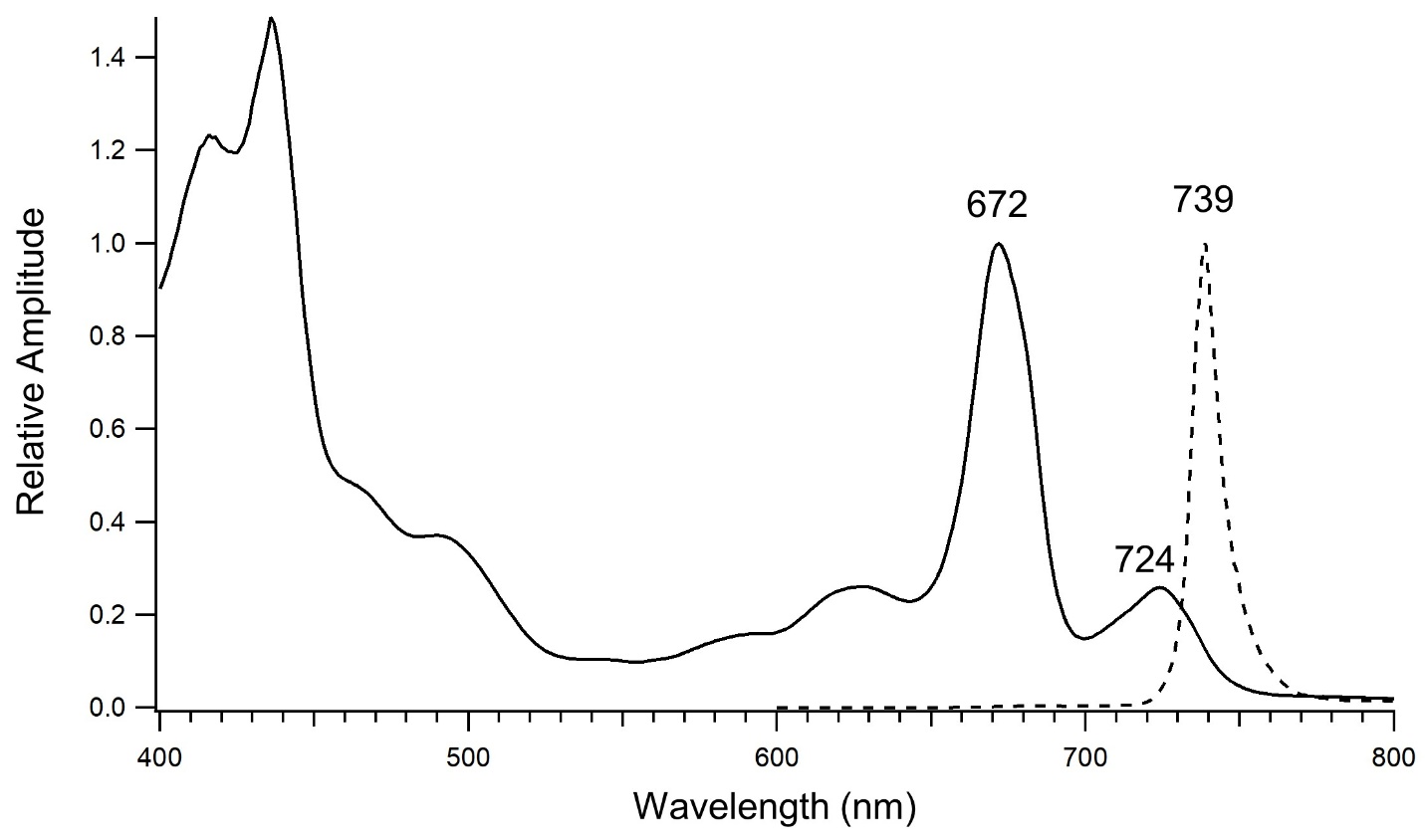


**Figure S1. Absorption and fluorescence emission spectra of dimeric FRL-PSII complex from *Synechococcus* 7335.** Absorbance (solid line) and the 77 K fluorescence emission (dashed line) spectra of the FRL-PSII particles purified by immobilized metal-affinity chromatography from FRL-grown cells of the *Synechococcus* sp. PCC 7335-*psbC2*-[His]_10_ strain. For comparison, spectra were normalized to 1.0 at the 672 nm absorbance maximum and the 739 nm fluorescence emission maximum.

**
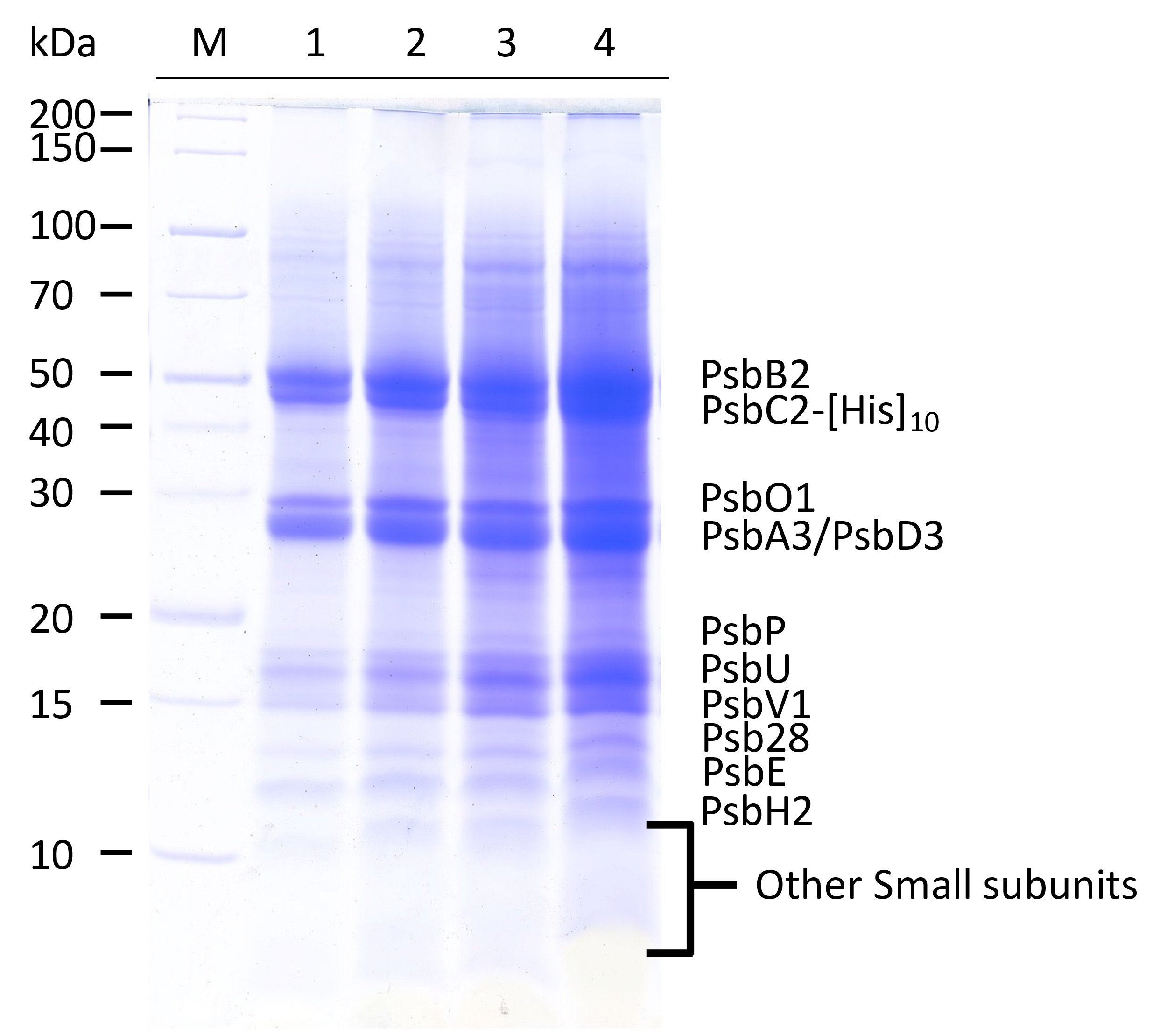
**

**Figure S2. SDS-PAGE analysis of the purified FRL-PSII complexes from *Synechococcus* 7335.** Proteins correspond to the following Chl *a* contents: Lane 1, 4 µg Chl; lane 2, 6 µg Chl; lane 3, 8 µg Chl, and lane 4, 10 µg Chl. Molecular mass markers (M) and their masses are indicated on the left.

**
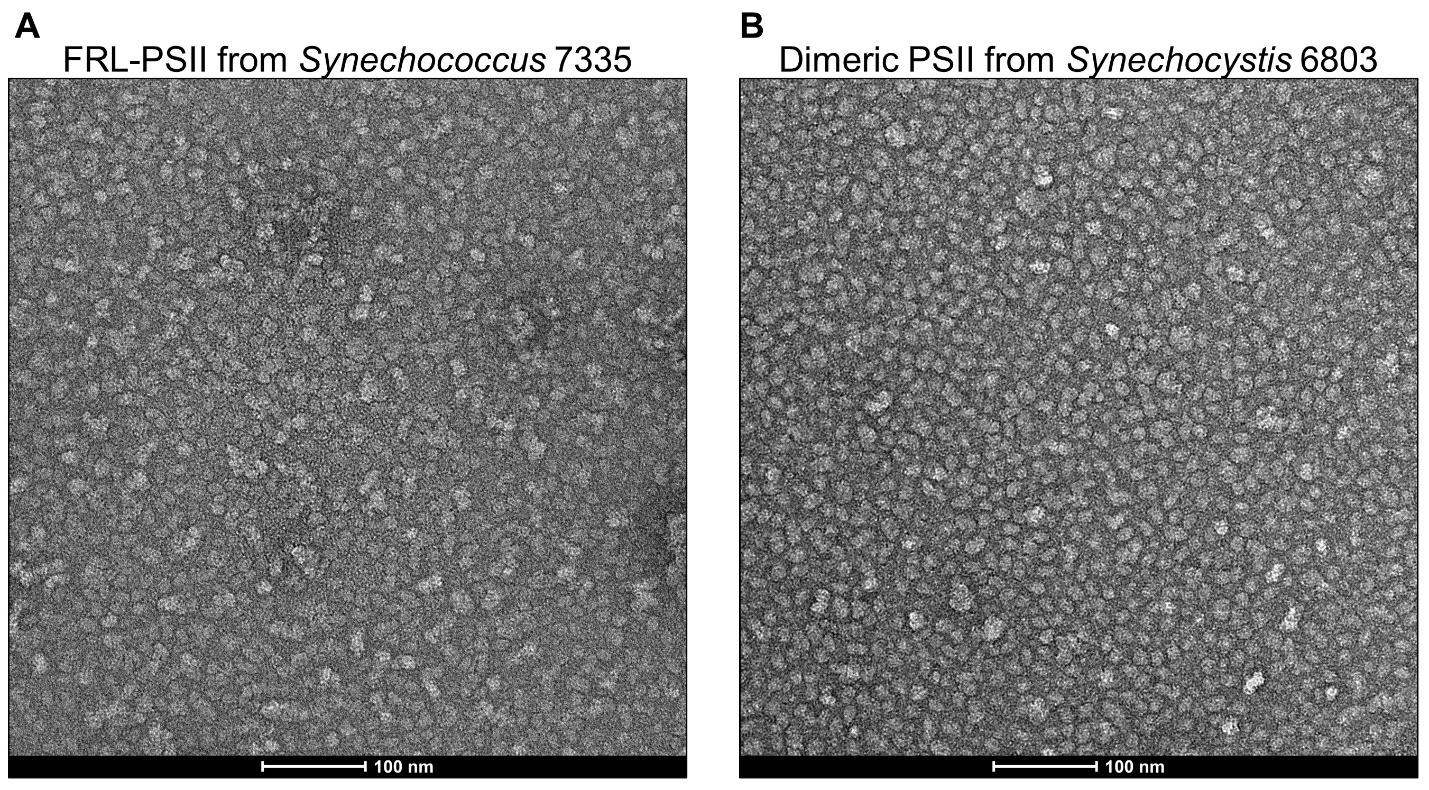
**

**Figure S3. Transmission electron microscopy of negatively stained FRL-PSII complexes from *Synechococcus* 7335 and comparison with dimeric PSII complexes from *Synechocystis* 6803. A.** FRL-PSII from *Synechococcus* 7335 at ~1 µg Chl mL^–1^. **B.** Dimeric PSII from *Synechocystis* 6803 at ~1 µg Chl mL^–1^.

**
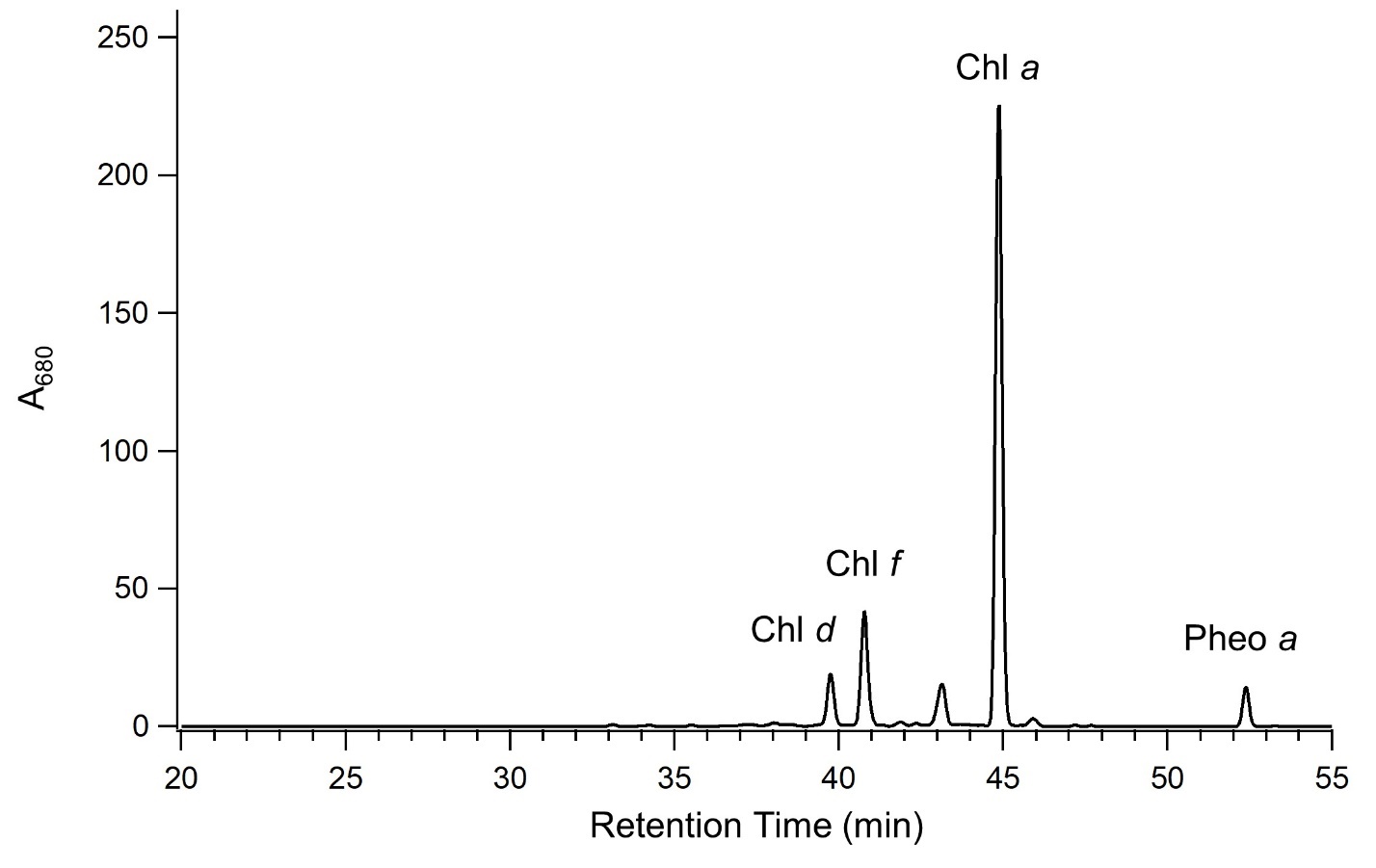
**

**Figure S4. Pigment analysis of the purified FRL-PSII complexes from *Synechococcus* 7335.** Pigments were extracted with acetone-methanol (7:2, v/v) and were analyzed using reversed-phase HPLC. The eluate was monitored at 680 nm. The small peak at 43 min had the same absorbance as Chl *a* and probably carried a more oxidized esterifying alcohol than phytol.

**
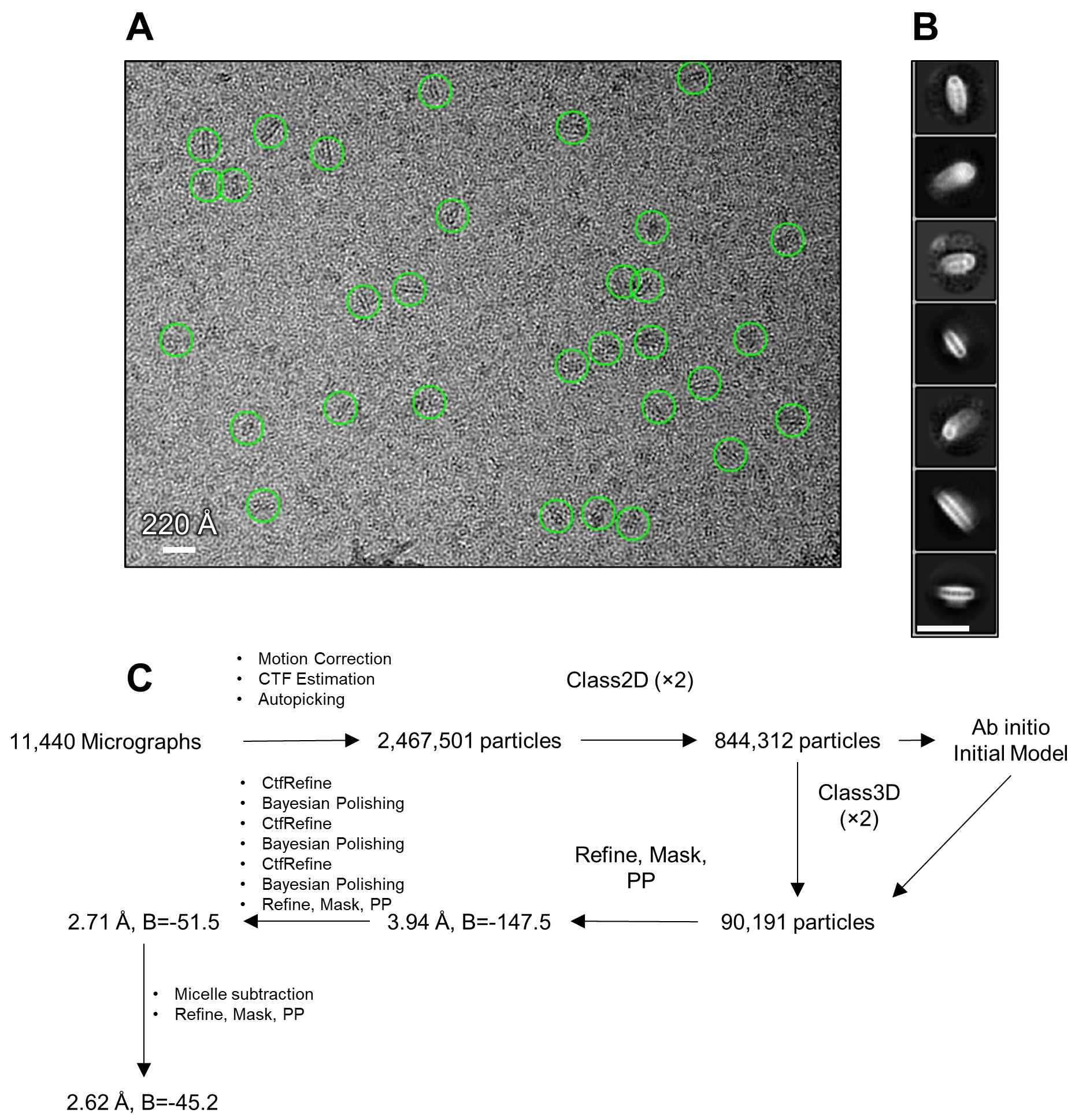
**

**Figure S5. Cryo-EM data and processing workflow for the dimeric FRL-PSII structure. A.** Example micrograph image with FRL-PSII particles circled. **B.** Example 2D classes. The scale bar corresponds to 200 Å. C. Data processing workflow in RELION 3.1 (50). PP = PostProcess job type. The B-factor is reported in units of Å^2^.

**
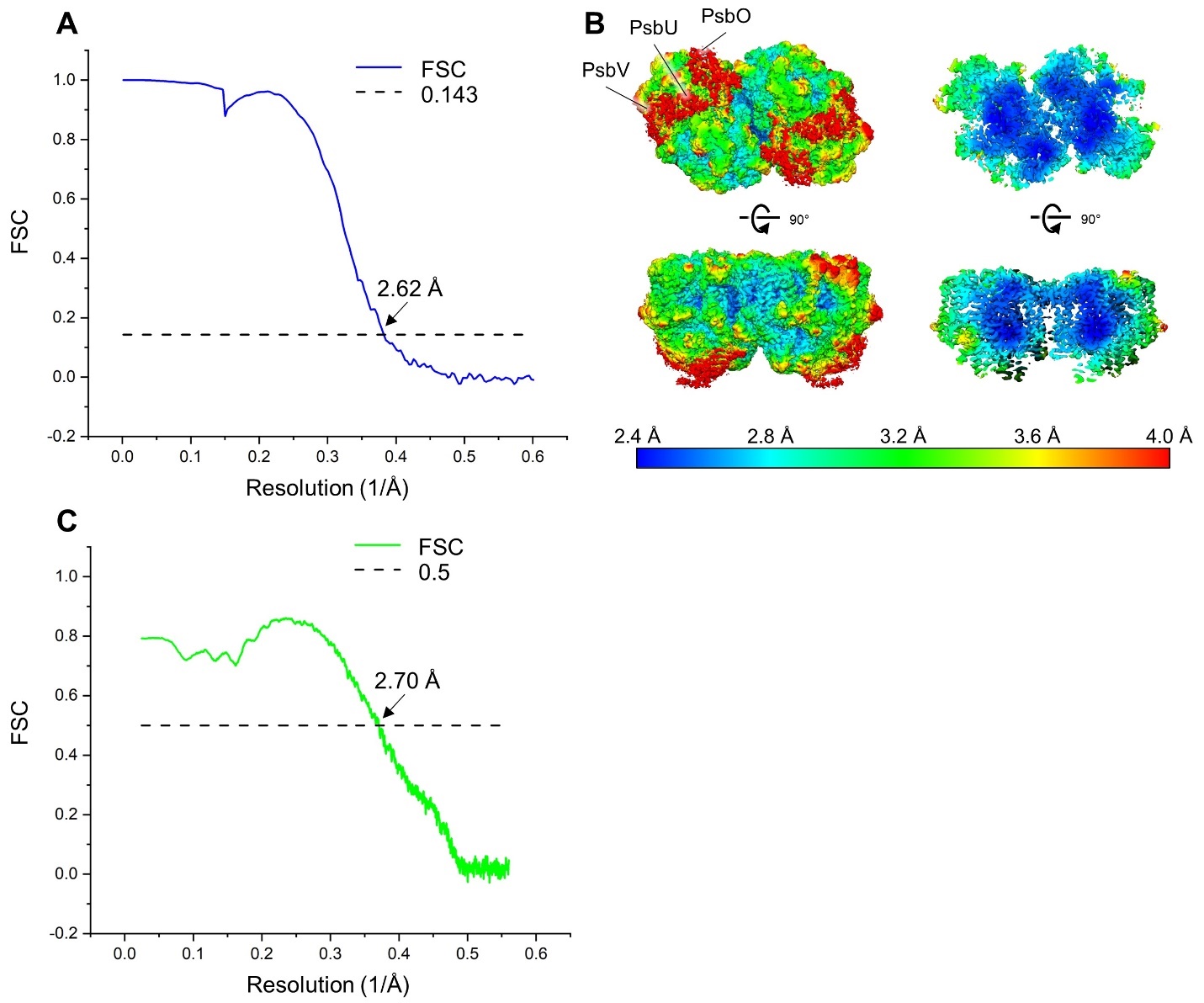
**

**Figure S6. Resolution of the FRL-PSII cryo-EM map. A.** Map-map Fourier shell correlation (FSC). The FSC value at 0.143 corresponds to 2.6-Å resolution. **B.** Local resolution map generated in RELION 3.1 (50). The unsharpened map is shown in two orientations showing the entire map (left) or a central slice of the map (right) and colored according to the key. Low occupancy extrinsic subunits are labeled. **C.** Map-model Fourier shell correlation generated in Phenix (61). The FSC value at 0.5 corresponds to 2.7-Å resolution.

**
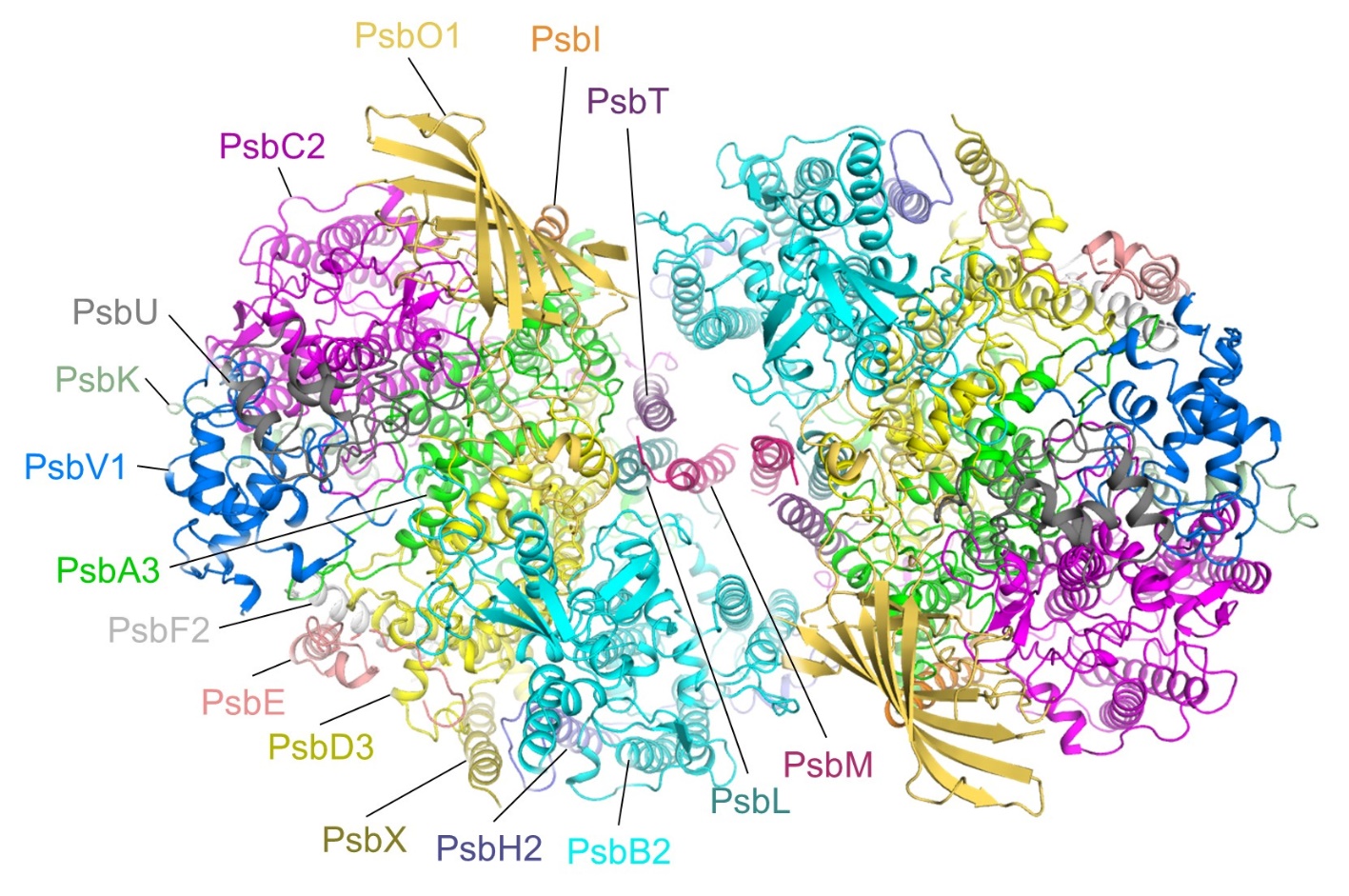
**

**Figure S7. Subunits modeled in the cryo-EM map.** Subunits are labeled individually on one of the two monomers in the dimeric FRL-PSII complex. The view is of the lumenal side.

**
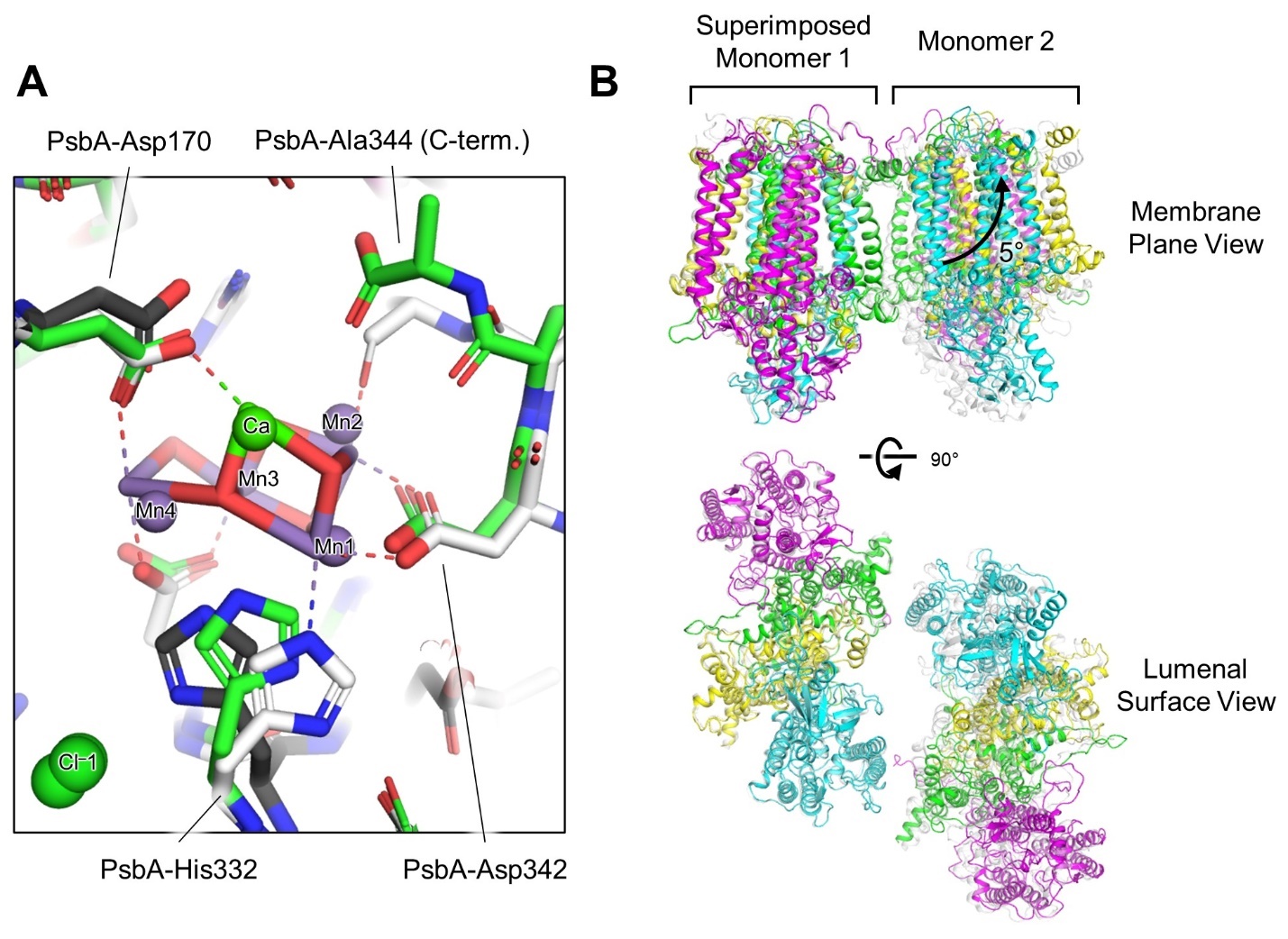
**

**Figure S8. Consequences of low occupancy extrinsic subunits. A.** OEC region comparing the dimeric FRL-PSII structure (carbon atoms are colored green), a PSII holocomplex structure from *Synechocystis* 6803 containing the OEC at high occupancy (carbons are colored white, PDB 7N8O), and an apo-PSII structure from *Synechocystis* 6803 lacking the OEC (carbons are colored dark grey, PDB 6WJ6). Stick representation is shown for the high occupancy OEC from the PSII holocomplex structure and sphere representation is shown for the lower occupancy OEC from the dimeric FRL-PSII complex. PsbA2-His332 is found in an intermediate position compared to the two *Synechocystis* 6803 PSII structures, implying low occupancy of the OEC. Furthermore, whereas the PsbA C-terminus ligates the OEC in the PSII holocomplex, it is shifted away in the dimeric FRL-PSII complex. In the apo-PSII complex, the PsbA C-terminus is not modeled and was presumed to be flexible (62). Note that for simplicity, the residue numbers used for labeling are consistent with those discussed frequently in other PSII literature. In PsbA2, the residue number is actually one more than is shown. For example, PsbA-His332 from *Synechocystis* 6803 is PsbA2-His333 in FRL-PSII. **B.** Superposition of core subunits (PsbA, PsbB, PsbC, and PsbD only are shown) in one monomer of the dimeric FRL-PSII structure (colored) with one monomer of PSII from *Synechocystis* 6803 (PDB 7N8O) (transparent grey). The comparison shows that in FRL-PSII, relative to monomer 1, monomer 2 is shifted ~5° about a hinge axis at the stromal side dimerization interface. This is most likely due to the loss of the extrinsic subunits on the lumenal side that are present at high occupancy in the *Synechocystis* 6803 PSII holocomplex.

>PsbO_EDX87597.1 Manganese-stabilizing protein / photosystem II polypeptide [Synechococcus sp. PCC 7335]

ACSGTKIAGDSLTYDDIRGSGLANDCPALTGTNMDAIPLEGSQPYQIRSLCLQPQTFLVETFPSVNGQLK

KQAGRFVEGKLLTRSSFTLDQVSGQLQKASNGALTFVEQGGFDFQPVTVQIPNGDRVPLLFTVKGLVAKT

AEAIDTIRPSTRFSGSFDVPPYRTSSFIDPKGRGLAVGYDAAVGIPIQADREAFSRQNNKSFKVGTGNIV

LQVDRVNQSTGEISGSFESQQPSDTDFGSKPAMTVKILGQFYSRITPEIA

>PsbU_WP_006455429.1 photosystem II complex extrinsic protein PsbU [Synechococcus sp. PCC 7335]

ASPSNSNTAFFANGQINGITLLANAGGKDLRNTVDDKLATAYGSKIDVNNTNIAAFRKHRGLYPTIAGKV

VSNAPYDSIEDILDIPGLREVEKDRLQKNMDIFTISDPVPALVEGADRFNNGVYK

>PsbV_WP_006453422.1 photosystem II cytochrome c-550 [Synechococcus sp. PCC 7335]

AAELDANLRTIPLNEAGDTVTLSLEEFTRGQQKFNNACAICHLGGITKTNPNVGLDTESLAGAFPDRNNL

EGLVDYLHNPTTYDGLTEISELHPSTKSSDIYPKMRNLTEDDLRAISGHILLMPKIRGDQWGAGKTKSL

**Figure S9. Modeled residues in the extrinsic subunits.** The sequences of PsbO1, PsbU, and PsbV1 from *Synechococcus* 7335 are shown. Residues not modeled in the structure are highlighted in red. Residues where only the backbone was modeled (i.e., the sidechain was trimmed) are highlighted in grey. Signal peptide residues have been omitted from the sequences.

**
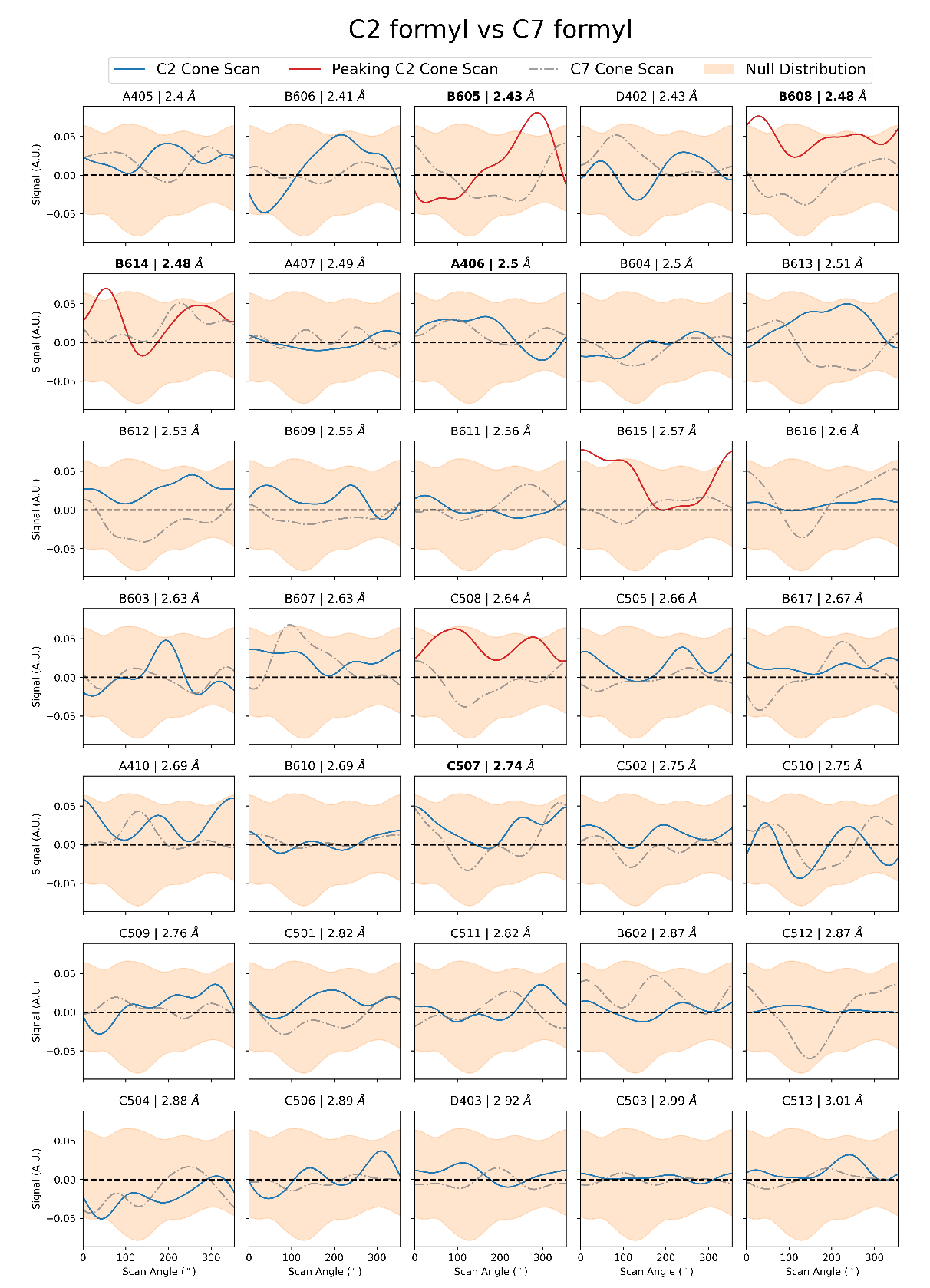
**

**Figure S10. Cone scans of Chl sites.** The cone scan of each Chl site is shown where the null distribution is shown in orange, the C2 scan is shown in blue or red, and the C7 scan (always methyl) is shown as a dashed line. Scans that have their C2 scan greater than the null distribution are shown with their C2 scan in red. Bold font refers to Chls that were assigned either Chl *d* or Chl *f* previously. The local resolution for each site is listed next to its site name. Formyl moiety bond geometry was used for this analysis.

**
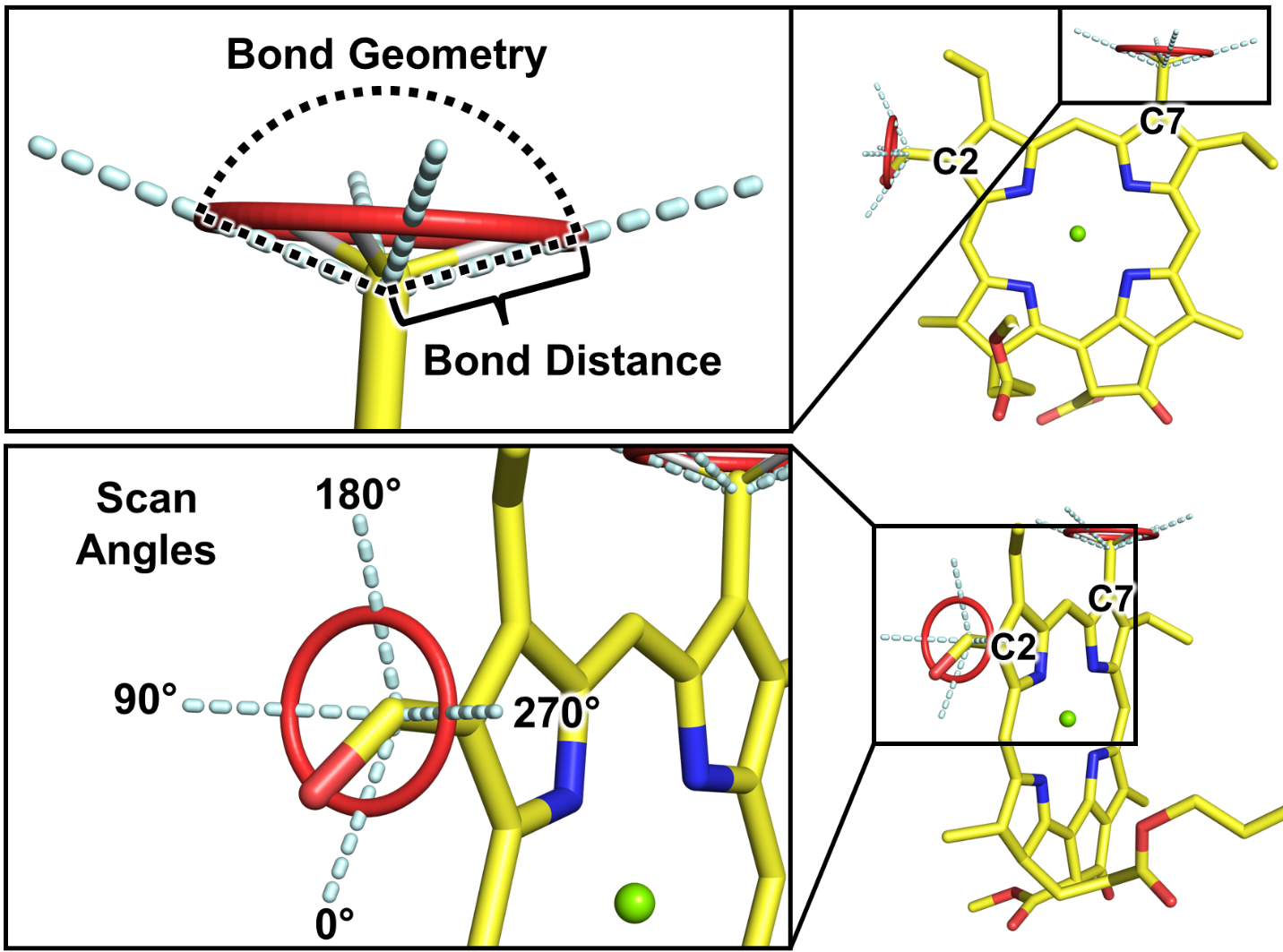
**

**Figure S11. Cone scan measurement diagram.** The figure shows a Chl *f* molecule in two views to depict the measurements involved in cone scan generation (**Fig. S10**) as described previously (22). The methyl null distribution is generated using cone scans at the C7 substituent using bond distance and geometry expected for a formyl moiety, that is a 1.23 Å bond distance and sp^2^ hybridization bond geometry. The cone scans at the C2 substituent are compared to the null distribution by plotting the cryo-EM map signal against the null distribution (**Fig. S10**). The cone scan angles used for plotting are shown as a dashed, pale-cyan lines while example cone scans are shown as solid, red rings.

**
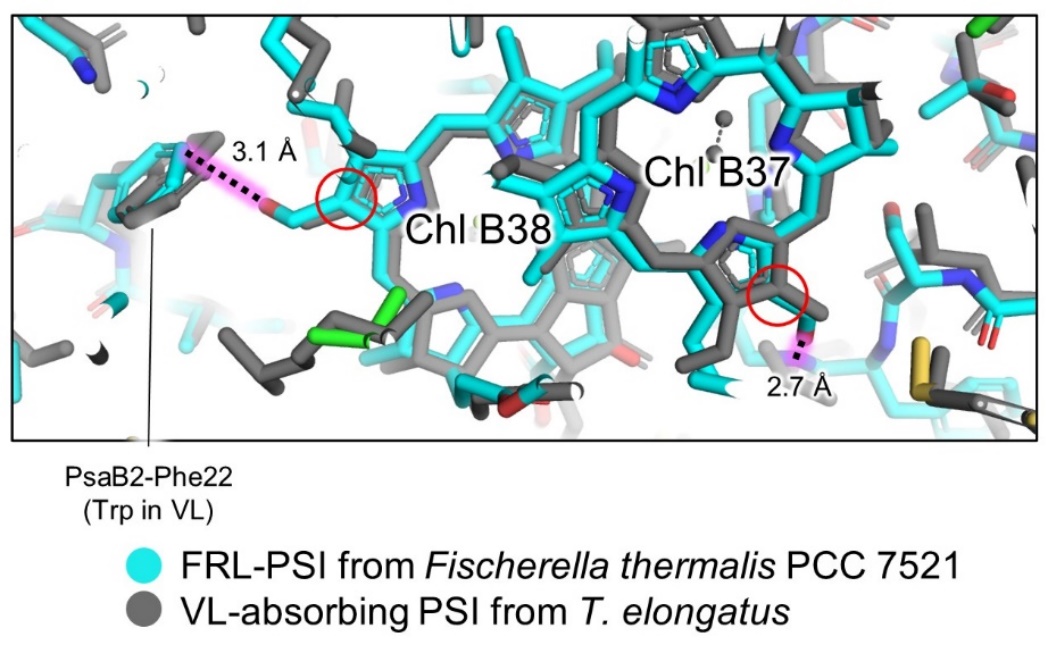
**

**Figure S12. OH–π interaction in position B38 of FRL-PSI.** The structure of FRL-PSI from *Fischerella thermalis* PCC 7521 (PDB 7LX0) is shown in colors superimposed with the structure of VL-absorbing PSI from *T. elongatus* (PDB 1JB0). This Chl dimer was proposed be two Chl *f* molecules (27). The C2 position of the tetrapyrrole ring of each Chl molecule is denoted with a red circle and FRL-specific interactions to the formyl moieties are highlighted in pink. Whereas the formyl moiety of Chl *f* in site B37 clearly has a FRL-specific H-bond donor from a backbone amide, the H-bond donor to the formyl moiety of Chl *f* in site B38 was unclear. We suggest that the FRL-specific Phe that replaces Trp found in non-FaRLiP and VL-PSI sequences confers specificity for Chl *f* at site B38 by a OH–π interaction with the conjugated system of the PsaB2-Phe22 sidechain.

**
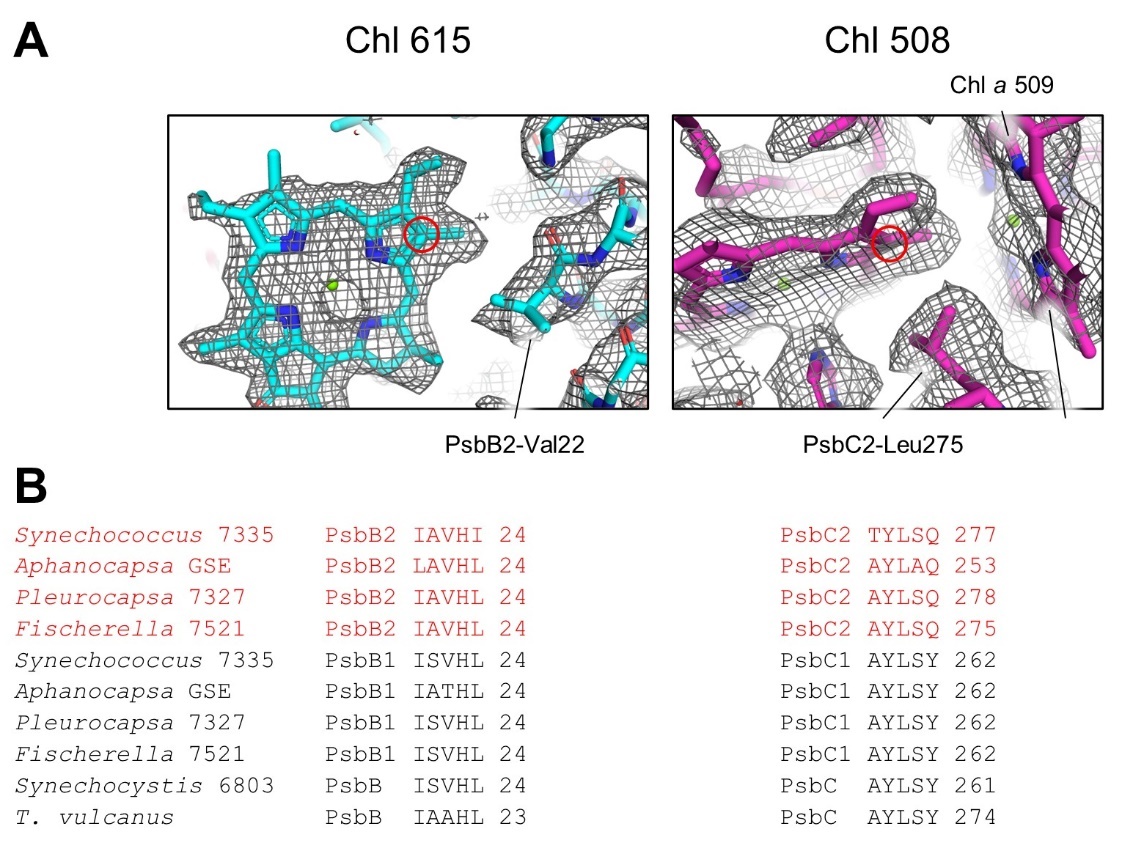
**

**Figure S13. False positive cone scan sites.** Two sites exhibited C2 cone scans higher than the methyl distribution that are unlikely to contain Chl *f*. **A.** The model within the map for each Chl site. Nearby residues/Chls that cause the false positive in the C2 cone scan (**Fig. S10**) are labeled. The C2 position of the tetrapyrrole ring of each Chl molecule is denoted with a red circle. **B.** Partial sequence alignment corresponding to panel **A**. FRL-specific sequences are shown in red font. Note that the residues labeled in **A** are not conserved in FRL sequences. In the partial sequence alignment of PsbC2, a Gln residue is FRL-specific, but this residue is located on the other side of the transmembrane helix that is shown, and it donates an H-bond to Chl *a* 505, which is adjacent to Chl *f* 507 as described previously (17).

**
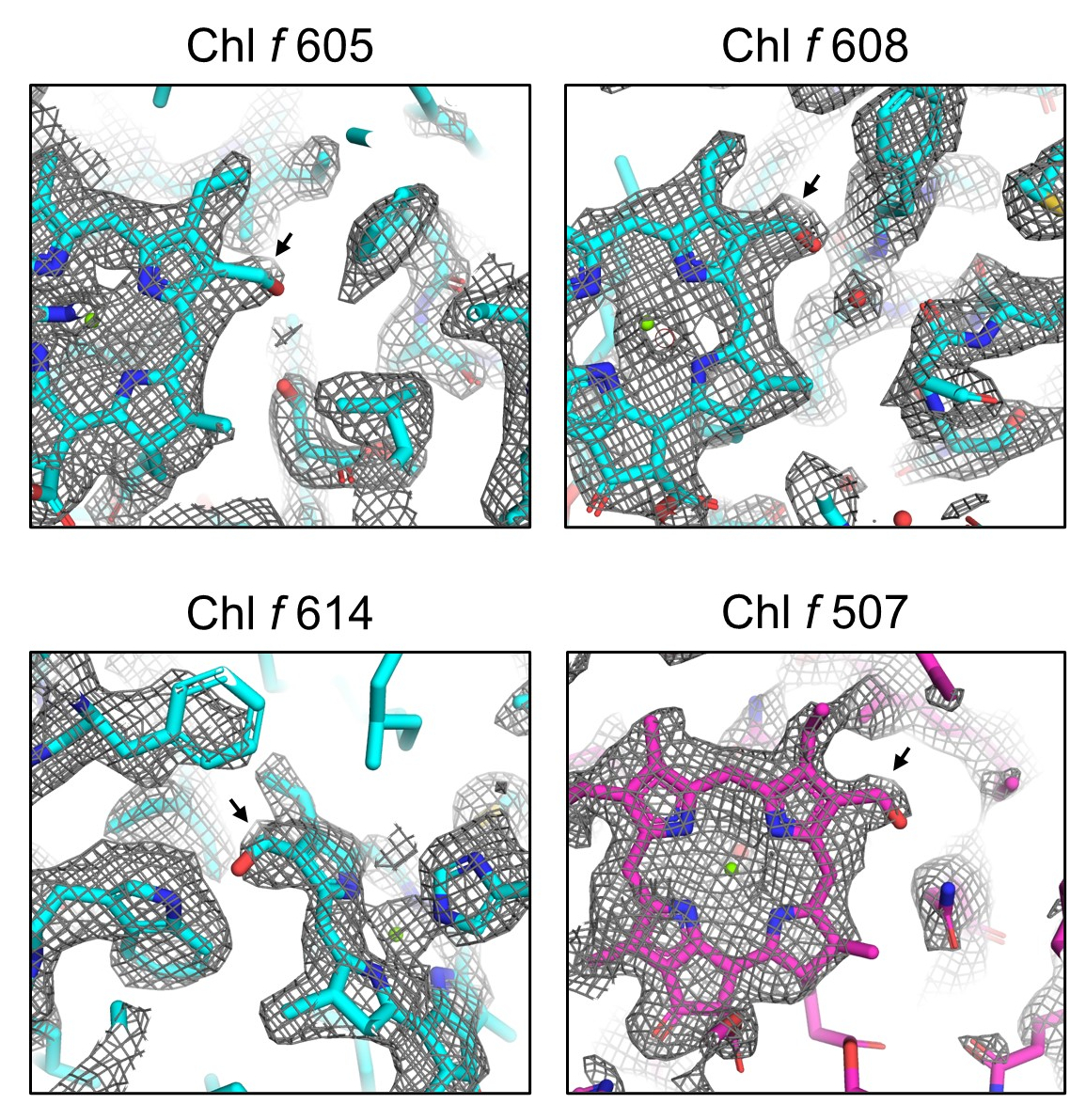
**

**Figure S14. Cryo-EM map regions for the Chl *f* assignments.** All maps are shown at 12σ. The C2 position is designated by a black arrow. This figure corresponds to **Fig. 2** where the protein interactions, corresponding sequence comparisons, and cone scans are shown.

**A** PsbH

FRL secondary structure hhhhh ttttttttttttttt

VL secondary structure hhhhhhhhhhhh ttttttttttttttt

**PsbH2 *Synechococcus* 7335 -------------MRQKYVSNKAAPLQYPLRKLNSEAGKVVPGWGTAPLMGIMLIALLLF 47**

PsbH2 *Aphanocapsa* GSE ------------MEQPNFPTKKVAPLQYFFRQFNSDAGKVVRGWGTAPLMVVLMLLFLVF 48

PsbH2 *Fischerella* 7521 ------------MQSQNFQPKKVAPVQYLLKQLNTEAGKVTPGWGTTPLMAALMLLLFIF 48

PsbH2 *Pleurocapsa* 7327 ------------MQVRKFAPKKVAPLQYFFKRFNSQAGKVIPGWGTTPLMLALMLLFFFF 48

PsbH1 *Synechococcus* 7335 -------------------MAQRTKLGNLLKPLNSEYGKVSPGWGTTPVMGVFMVLFLVF 41

PsbH1 *Aphanocapsa* GSE -------------------MAKQTWLGDVLRPLNAEYGKVAPGWGTTPLMAVFMGLFLVF 41

PsbH1 *Fischerella* 7521 -------------------MAQRTRLGDILRPLNSEYGKVAPGWGTTPLMAVFMGLFFVF 41

PsbH1 *Pleurocapsa* 7327 -------------------MAQRTRLGDILRPLNSEYGKVVPGWGTTPLMGVFMLLFLVF 41

**PsbH *Synechocystis* 6803 -------------------MAQRTRLGDILRPLNSEYGKVVPGWGTTPVMGVFMALFLVF 41**

**PsbH *T. vulcanus* --------------------ARRTWLGDILRPLNSEYGKVAPGWGTTPLMAVFMGLFLVF 40**

**PsbH *Arabidopsis* MA-------TQTVEDSSRSGPRSTTVGKLLKPLNSEYGKVAPGWGTTPLMGVAMALFAVF 53**

**PsbH *Chlamydomonas* MATGTSKAKPSKVNSDFQEPGLVTPLGTLLRPLNSEAGKVLPGWGTTVLMAVFILLFAAF 60**

**PsbH *Pisum* MA-------TQTVENSSRSGPRRTAVGDLLKPLNSEYGKVAPGWGTTPLMGIAMALFAVF 53**

: : :: :*:: *** ****: :* : : *

FRL secondary structure ttttttttt

VL secondary structure ttttttttt

**PsbH2 *Synechococcus* 7335 ILTILQLYNGTVIVEGIDV----------------------------------------- 66**

PsbH2 *Aphanocapsa* GSE LLIILQIFNASILLEGINIDWSTLSDYAEVAASTPYGQGQFASTGTSIFIGLVVFALTCL 108

PsbH2 *Fischerella* 7521 LLMILEIVNSSILLEGINMS---------------------------------------- 68

PsbH2 *Pleurocapsa* 7327 LLMLLEIVNASIQLEGIDVDWKSLSY---------------------------------- 74

PsbH1 *Synechococcus* 7335 LLIILQLYNSSLIINNVDVDWRSLGN---------------------------------- 67

PsbH1 *Aphanocapsa* GSE LLIILQIYNSSLILDGVNVTWKSFAG---------------------------------- 67

PsbH1 *Fischerella* 7521 LLIILQLYNKSIIIQDVSVDWRSLGR---------------------------------- 67

PsbH1 *Pleurocapsa* 7327 LLIILQIYNSSLLLEGFSVDWRSLGQ---------------------------------- 67

**PsbH *Synechocystis* 6803 LLIILQIYNSSLILEGFSVDWAG------------------------------------- 64**

**PsbH *T. vulcanus* LLIILEIYNSTLILDGVNVSWKALG----------------------------------- 65**

**PsbH *Arabidopsis* LSIILEIYNSSVLLDGISVN---------------------------------------- 73**

**PsbH *Chlamydomonas* LLIILEIYNSSLILDDVSMSWETLAKVS-------------------------------- 88**

**PsbH *Pisum* LSIILEIYNSSLLLDQISMN---------------------------------------- 73**

: :*:: * :: :: ..:

FRL secondary structure

VL secondary structure

**PsbH2 *Synechococcus* 7335 --------------- 66**

PsbH2 *Aphanocapsa* GSE ALIFYGATTYPKDQR 123

PsbH2 *Fischerella* 7521 --------------- 68

PsbH2 *Pleurocapsa* 7327 --------------- 74

PsbH1 *Synechococcus* 7335 --------------- 67

PsbH1 *Aphanocapsa* GSE --------------- 67

PsbH1 *Fischerella* 7521 --------------- 67

PsbH1 *Pleurocapsa* 7327 --------------- 67

**PsbH *Synechocystis* 6803 --------------- 64**

**PsbH *T. vulcanus* --------------- 65**

**PsbH *Arabidopsis* --------------- 73**

**PsbH *Chlamydomonas* --------------- 88**

**PsbH *Pisum* --------------- 73**

**B** PsbB (partial)

Residues unmodeled in the apo-FRL-PSII structures: ||||||||| ||||

**PsbB2 *Synechococcus* 7335 WTFETVAIAHIIFSGLSFLAACWHWVYWDVATFFDPKTD-----------EPVIDLPKVF 139**

PsbB2 *Aphanocapsa* GSE WTLESVAIAHIIFSGLEFLAAVWHWNYWNISTFFDPKTN-----------KPILDLPRIF 139

PsbB2 *Fischerella* 7521 WTFETVAIAHIVFSGLEFLAACWHWFYWDLATFFDSKTG-----------EPTLDLPKIF 139

PsbB2 *Pleurocapsa* 7327 WTFETVAIAHIVFSGLEFLAAIWHWVNWDLVTFFDEKTG-----------EPTLDLPRIF 139

PsbB1 *Synechococcus* 7335 WSFEGVALAHIVLSGLLFLAACWHWVFWDLELFRDPRTG-----------EPALDLPKMF 139

PsbB1 *Aphanocapsa* GSE WSFEGVALAHIVLSGLLFLAAIWHWVNWDLELFRDPRTG-----------EPALDLPKMF 139

PsbB1 *Fischerella* 7521 WSFEGVAAAHIVLSGLLFLAAVWHWVYWDLELFQDPRTG-----------EPALDLPKMF 139

PsbB1 *Pleurocapsa* 7327 WSFEGVAAAHIVLSGLLFLAAVWHWVYWDLELFTDPRTG-----------EPALDLPKMF 139

**PsbB *Synechocystis* 6803 WSFEGVAAAHIVLSGLLFLAAVWHWVFWDLELFVDPRTG-----------ESALDLPKMF 139**

**PsbB *T. vulcanus* WSFEGVALAHIVLSGLLFLAACWHWVYWDLELFRDPRTG-----------EPALDLPKMF 138**

**PsbB *Arabidopsis* --YFVSGVLHLISSAVLGFGGIYHALL-------GPETLEESFPFFGYVWKDRNKMTTIL 161**

**PsbB *Chlamydomonas* --YFVSGVLHLISSAVLGFGGVYHSLI-------GPETLEESYPFFGYVWKDKNKMTNIL 149**

**PsbB *Pisum* --YFVSGVLHLISSAVLGFGGIYHALL-------GPETLEESFPFFGYVWKDRNKMTTIL 161**

. *:: *.: :.. :* . .* : .: ::

**Figure S15. Multiple sequence alignment of PsbH and partial sequence alignment of PsbB.** Structures are available for sequences in bold font. Sequences in red font are FRL-specific isoforms. Sequences in black font are VL-specific isoforms or non-FaRLiP sequences. Residues highlighted in magenta correspond to the N-terminal extension found in FRL-specific sequences. Residues highlighted in yellow are those conserved in all FRL sequences determined previously (17). Sequences were aligned using Clustal Omega (32). **A.** Multiple sequence alignment of PsbH. Secondary structure is shown above the alignment for FRL and VL/non-FaRLiP cyanobacterial structures, respectively. Whereas the residues composing the transmembrane α-helix (*t*) are aligned, the α-helix on the stromal surface (*h*) near the N-terminus is shorter in FRL sequences. **B.** Partial sequence alignment of PsbB focusing on the region that was unresolved in the apo-FRL-PSII structure reported previously (18). Lines above the alignment signify the residues that were not resolved in the apo-FRL-PSII structure.


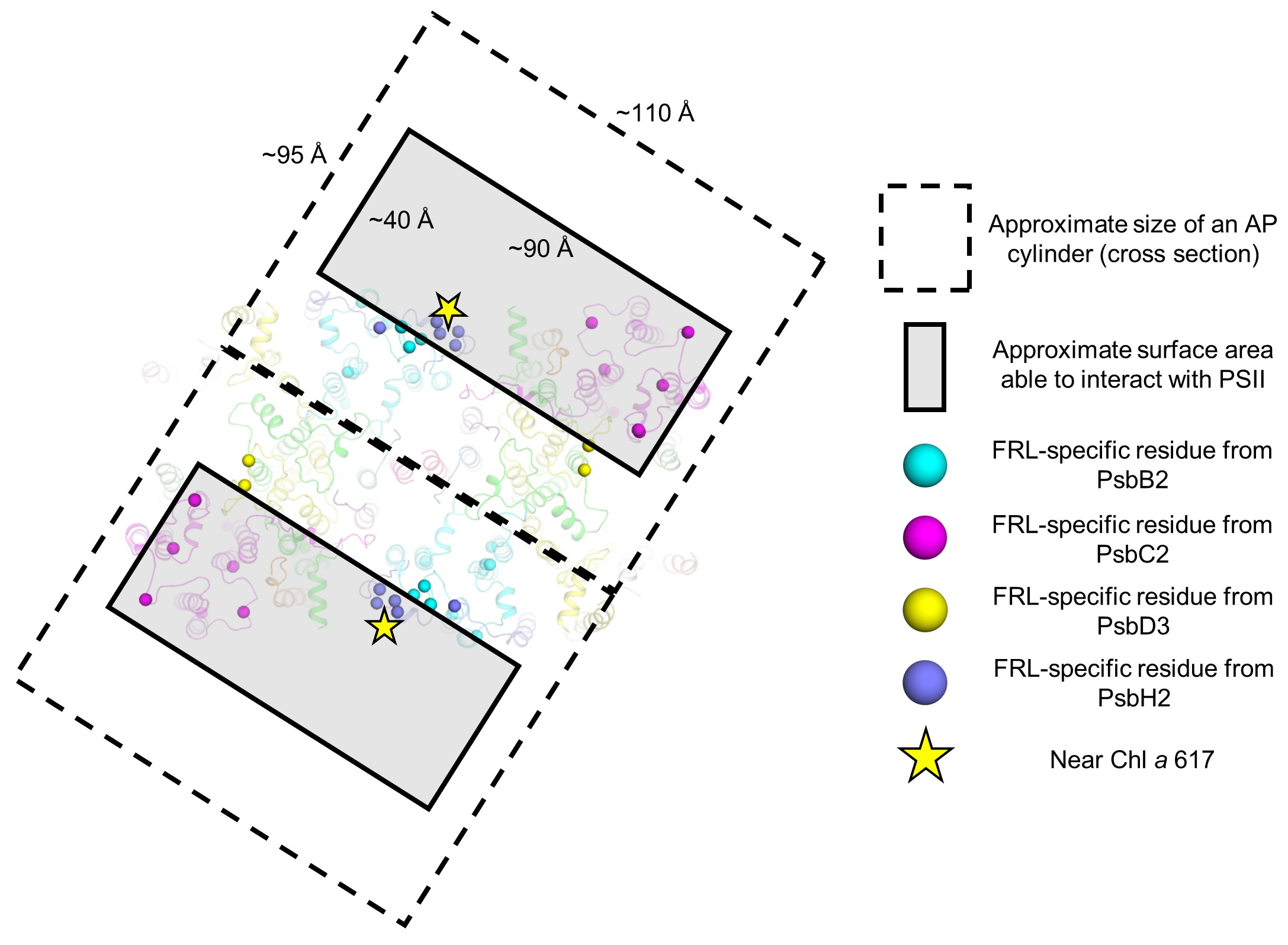


**Figure S16. FRL-specific PSII residues on the stromal side and suggested region of FRL-phycobiliprotein complex binding to FRL-PSII.** The diagram represents a stromal view of a FRL-PSII dimer where each PSII monomer is colored differently. Each monomer has two sides containing the labeled subunits. Other subunits are excluded for clarity. The proposed region of the FRL-phycobiliprotein complex is shown with a dashed line (AP = allophycocyanin). PsbA3 is not listed in the key because there are no FRL-specific residues from PsbA3 found on the stromal surface.

**
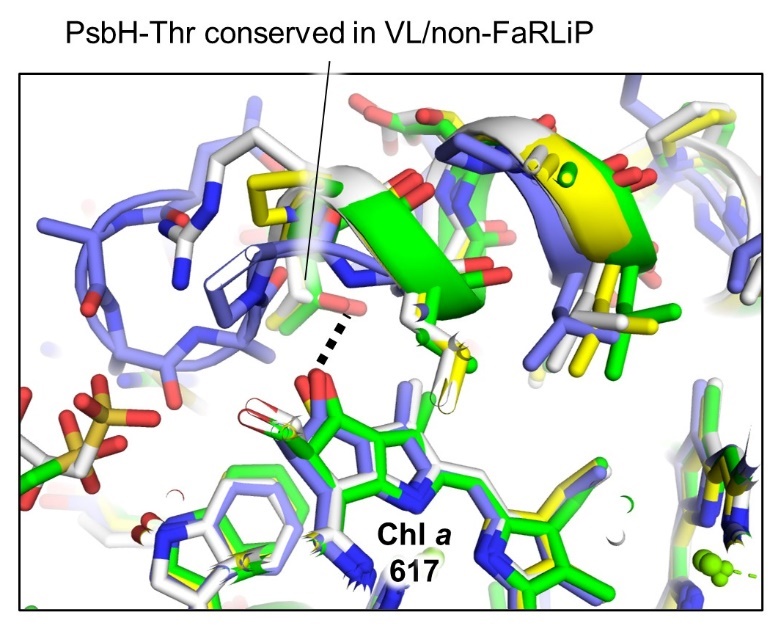
**

**Figure S17. Structural conservation of PsbH-Thr H-bonding with Chl *a* 617.** The Thr sidechain conserved in PsbH sequences from VL sequences and non-FaRLiP sequences is labeled, and the H-bond is shown with a dashed line. The structure of the dimeric FRL-PSII complex from *Synechococcus* 7335 (blue, cyanobacterial) is shown superimposed with PSII structures from *Synechocystis* 6803 (white, cyanobacterial) (26), *Pisum sativum* (green, higher plant) (71), and *Chlamydomonas reinhardtii* (yellow, green algal) (72).

**
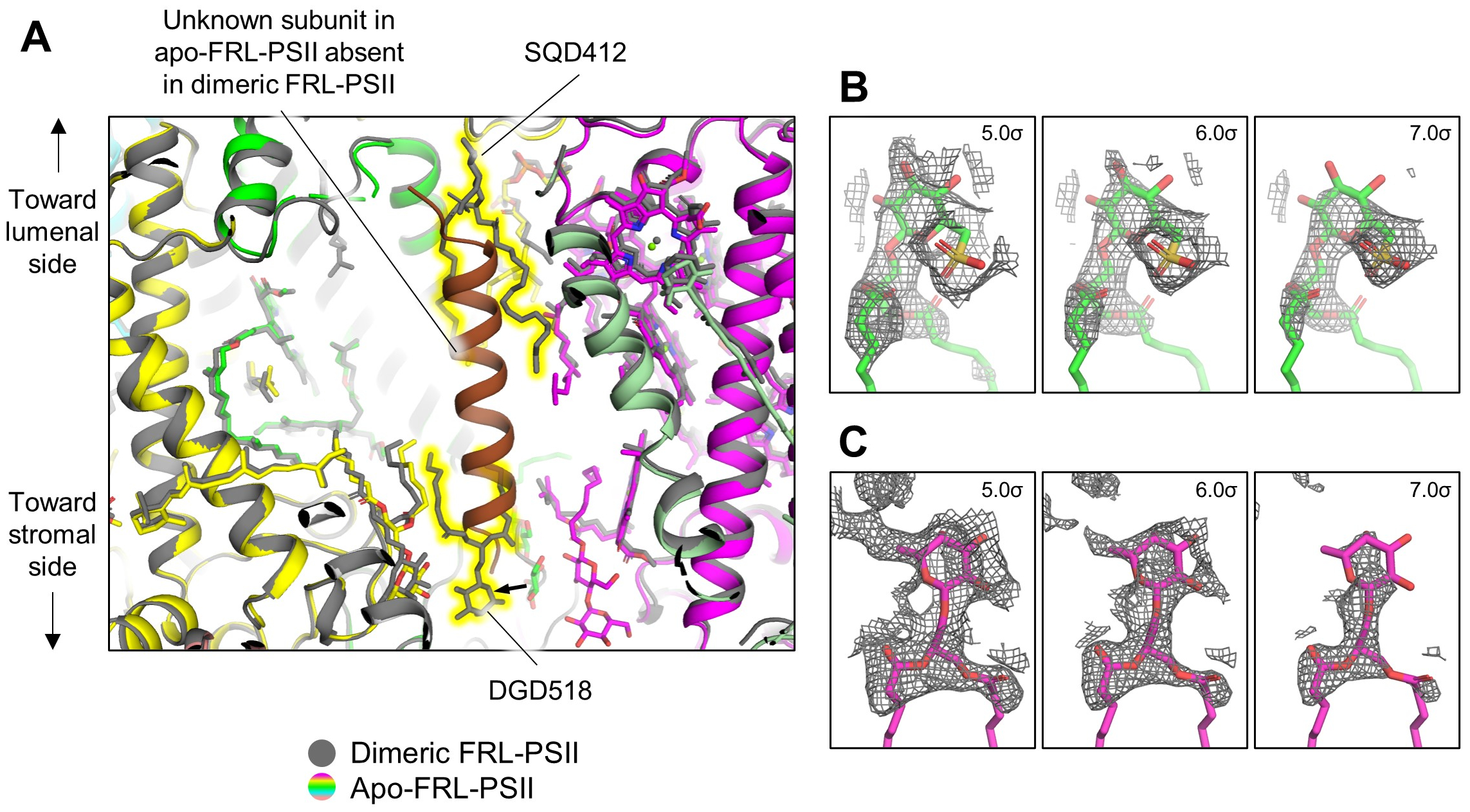
**

**Figure S18. Lipids occupying the region of the dimeric FRL-PSII structure in the site where the unknown subunit was present in the apo-FRL-PSII structure.** **A.** The apo-FRL-PSII (colored) and dimeric FRL-PSII (grey) are superimposed as shown in **Fig. 4**. The view shown is from within the membrane. The unknown transmembrane subunit observed in the apo-FRL-PSII structure is shown (brown), and the two lipids that occupy that region in the dimeric FRL-PSII structure are shown (highlighted in yellow). Relative to the apo-FRL-PSII structure, the dimeric FRL-PSII structure shows the DGD518 lipid shifted into the site where the unknown subunit had been modeled (small black arrow). A lipid analogous to SQD412 on the lumenal side is not found in the apo-FRL-PSII structure but is conserved in other dimeric PSII structures. **B.** The unsharpened cryo-EM map and model for SQD412 at 5.0σ, 6.0σ, and 7σ. **C.** The unsharpened cryo-EM map and model for DGD518 at 5.0σ, 6.0σ, and 7σ.

**Supporting Tables**

**Table S1. Proteins identified by tryptic peptide fingerprinting and MS/MS mass spectrometry of the FRL-PSII isolation.** Some minor contaminating proteins not associated with PSII were not included. Proteins encoded by the FaRLiP gene cluster are shaded in pink. Proteins in bold were observed in the cryo-EM map as subunits of FRL-PSII.

| **Protein** | **–10lgP** | **% Coverage** | **#Peptides** | **#Unique** | **#Spec** | **Description** |
| --- | --- | --- | --- | --- | --- | --- |
| **PsbC2** | **327.62** | **52** | **79** | **76** | **141** | **S7335_3753 photosystem II CP43 reaction center protein** |
| **PsbO1** | **318.53** | **55** | **53** | **53** | **122** | **S7335_5307 photosystem II manganese-stabilizing protein** |
| **PsbB2** | **308.89** | **52** | **68** | **68** | **136** | **S7335_4830 photosystem II CP47 reaction center protein** |
| **PsbA3** | **305.05** | **34** | **34** | **32** | **78** | **S7335_4273 photosystem II D1 reaction center protein** |
| **PsbD3** | **280.85** | **40** | **45** | **31** | **128** | **S7335_1444 photosystem II D2 reaction center protein** |
| **PsbE** | **253.17** | **66** | **22** | **22** | **40** | **S7335_3478 cytochrome *b*_559_ alpha subunit** |
| **PsbU** | **245.42** | **42** | **24** | **24** | **38** | **S7335_3378 photosystem II 12 kDa extrinsic protein** |
| PsbD1 | 209.28 | 11 | 15 | 1 | 33 | S7335_1444 photosystem II D2 protein |
| **PsbV1** | **178.63** | **39** | **15** | **15** | **23** | **S7335_4926 photosystem II cytochrome *c*_550_** |
| PsbP | 159.30 | 14 | 5 | 5 | 12 | S7335_1471 photosystem II protein PsbP |
| **PsbH2** | **159.06** | **61** | **11** | **11** | **16** | **S7335_1601 photosystem II 10 kDa protein** |
| PsbA1 | 136.47 | 16 | 6 | 4 | 7 | S7335_2623 photosystem II D1 protein (also S7335_1528) |
| PsbA2 | 136.47 | 15 | 6 | 4 | 7 | S7335_0157 photosystem II D1 protein |
| Psb28 | 125.75 | 37 | 8 | 8 | 14 | S7335_2036 photosystem II reaction center Psb28 protein |
| PsbC1 | 123.17 | 12 | 6 | 4 | 8 | S7335_4424 photosystem II CP43 reaction center protein |
| **PsbF2** | **108.07** | **57** | **4** | **4** | **4** | **S7335_3212 cytochrome *b*_559_ beta subunit** |
| **PsbX** | **101.16** | **49** | **5** | **5** | **11** | **S7335_4754 photosystem reaction center X** |
| Ycf12 | 90.26 | 28 | 1 | 1 | 2 | S7335_1519 photosystem II reaction center protein Ycf12 |
| PsbF1 | 77.21 | 32 | 2 | 2 | 2 | S7335_3021 cytochrome *b*_559_ beta subunit |
| PsbO2 | 58.53 | 5 | 1 | 1 | 1 | S7335_4784 photosystem II manganese-stabilizing protein |
| Ycf48 | 45.81 | 5 | 1 | 1 | 1 | S7335_3142 photosystem II assembly factor Ycf48 |
| PsbV2 | 40.82 | 15 | 2 | 2 | 2 | S7335_1375 photosystem II cytochrome *c*_550_ |

**Table S2.** **Cryo-EM data statistics for the dimeric FRL-PSII complex.**

| **Data collection and processing** |  |  |
| --- | --- | --- |
| Magnification | ×105,000 | |
| Voltage (kV) | 300 | |
| Electron exposure (e^-^Å^-2^) | 41.1 | |
| Defocus range (µm) | –1.2 to –2.2 | |
| Pixel size (Å) | 0.416 | |
| Symmetry imposed | C2 | |
| Initial particle images (no.) | 2,467,501 | |
| Final particle images (no.) | 90,191 | |
| Map resolution (Å) | 2.6 | |
| FSC threshold | 0.143 | |
| **Refinement** |  | |
| Initial model used (PDB code) | 7SA3 and 7N8O | |
| Model resolution (Å) | 2.7 | |
| FSC threshold | 0.5 | |
| Map resolution range (Å) | 2.4-4.0 | |
| Map-sharpening *B* factor (Å^2^) | –45.2 | |
| Model composition |  | |
| Non-hydrogen atoms | 42,504 | |
| Protein residues | 4,678 | |
| Non-water ligands | 132 | |
| Waters | 590 | |
| *B* factors (Å^2^) |  | |
| Protein | 44 | |
| Ligands | 33 | |
| R.m.s. deviations |  | |
| Bond lengths (Å) | 0.014 | |
| Bond angles (°) | 1.82 | |
| **Validation** |  | |
| MolProbity | 2.26 | |
| Clashscore | 17.82 | |
| Rotamer outliers (%) | 1.39 | |
| Ramachandran plot |  | |
| Favored (%) | 93.98 | |
| Allowed (%) | 5.93 | |
| Disallowed (%) | 0.09 | |

**Table S3.** **Metal-metal distances in the OEC of PSII structures.** OEC metal-metal distances are reported in units of Å. For the *Synechocystis* 6803 (26) and *T. vulcanus* (25) PSII holocomplex structures, the difference compared to the dimeric FRL-PSII structure is additionally shown in parentheses.

|  | ***Synechococcus sp. PCC 7335***  **FRL-PSII Reported here** | ***Synechocystis* sp. PCC 6803**  **PSII holocomplex, PDB 7N8O** | ***Thermosynechcoccus vulcanus***  **PSII holocomplex, PDB 3WU2** |
| --- | --- | --- | --- |
| **Mn1-Ca** | 3.98 | 3.76 (-0.22) | 3.55 (-0.43) |
| **Mn1-Mn2** | 3.17 | 3.13 (-0.04) | 2.87 (-0.30) |
| **Mn1-Mn3** | 3.24 | 3.41 (+0.17) | 3.28 (+0.04) |
| **Mn1-Mn4** | 4.56 | 5.07 (+0.51) | 4.99 (+0.43) |
| **Mn2-Ca** | 3.48 | 3.58 (+0.10) | 3.39 (-0.09) |
| **Mn2-Mn3** | 3.04 | 3.05 (+0.01) | 2.88 (-0.16) |
| **Mn2-Mn4** | 5.37 | 5.66 (+0.29) | 5.44 (+0.07) |
| **Mn3-Ca** | 3.55 | 3.42 (-0.13) | 3.42 (-0.13) |
| **Mn3-Mn4** | 3.13 | 2.95 (-0.18) | 2.97 (-0.16) |
| **Mn4-Ca** | 3.31 | 3.86 (+0.55) | 3.79 (+0.48) |

**Table S4. Automated H-bond search for Chl *b* in LHCII.** In the “Known Chl type” column, cells are shaded blue if they correspond to Chl *b*. In the H-bond detection column, cells are shaded green if the search agrees with “Known Chl type” and red they do not.

| **Chl site** | **Known Chl type** | **H-bond donor detected near C7^1^ suggesting Chl *b*?** |
| --- | --- | --- |
| 601 | *a* | N |
| 602 | *a* | N |
| 603 | *a* | N |
| 604 | *a* | N |
| 605 | *a* | N |
| 606 | *a* | N |
| 607 | *a* | N |
| 608 | *a* | N |
| 609 | *b* | N |
| 610 | *b* | Y |
| 611 | *b* | Y |
| 612 | *b* | Y |
| 613 | *b* | Y |
| 614 | *b* | Y |

**Table S5. Automated H-bond search for Chls *d* and *f* in the dimeric FRL-PSII structure.** In the “Known Chl type” column, cells are shaded tan if they correspond to Chl *d* and pink they correspond to Chl *f*. In the H-bond detection columns, cells are shaded green if the search agrees with “Known Chl type” and red if they do not.

| **Chain** | **Chl site** | **Known Chl type** | **H-bond donor detected near C3^1^ suggesting Chl *d*?** | **H-bond donor detected near C2^1^ suggesting Chl *f*?** |
| --- | --- | --- | --- | --- |
| D | 402 (P_D2_) | *a* | N | N |
| D | 403 | *a* | N | N |
| A | 405 (P_D1_) | *a* | N | N |
| A | 406 (Chl_D1_) | *d* | Y | N |
| A | 407 (Chl_D2_) | *a* | N | N |
| A | 410 | *a* | N | N |
| B | 602 | *a* | N | N |
| B | 603 | *a* | N | N |
| B | 604 | *a* | N | N |
| B | 605 | *f* | N | N |
| B | 606 | *a* | N | N |
| B | 607 | *a* | N | N |
| B | 608 | *f* | N | Y |
| B | 609 | *a* | N | N |
| B | 610 | *a* | N | N |
| B | 611 | *a* | N | N |
| B | 612 | *a* | Y | N |
| B | 613 | *a* | N | N |
| B | 614 | *f* | N | Y |
| B | 615 | *a* | N | N |
| B | 616 | *a* | N | N |
| B | 617 | *a* | N | N |
| C | 501 | *a* | N | N |
| C | 502 | *a* | N | N |
| C | 503 | *a* | N | N |
| C | 504 | *a* | N | N |
| C | 505 | *a* | N | N |
| C | 506 | *a* | N | N |
| C | 507 | *f* | Y | Y |
| C | 508 | *a* | N | N |
| C | 509 | *a* | Y | N |
| C | 510 | *a* | N | N |
| C | 511 | *a* | N | N |
| C | 512 | *a* | N | N |
| C | 513 | *a* | N | N |

**Table S6. Sequence identity of PsbH2 with PsbH from two representative non-FaRLiP cyanobacteria.** Values are reported in units of %. The FRL-specific sequence is in red font.

|  | **PsbH2 from *Synechococcus* 7335** | **PsbH1 from *Synechococcus* 7335** | **PsbH from *Synechocystis* 6803** | **PsbH from**  ***T. vulcanus*** |
| --- | --- | --- | --- | --- |
| **PsbH2 from *Synechococcus* 7335** | 100.0 | 48.33 | 51.67 | 49.15 |
| **PsbH1 from *Synechococcus* 7335** | 48.33 | 100.0 | 78.12 | 70.77 |
| **PsbH from *Synechocystis* 6803** | 51.67 | 78.12 | 100.0 | 77.78 |
| **PsbH from *T. vulcanus*** | 49.15 | 70.77 | 77.78 | 100.0 |

**Table S7. C_α_ superposition RMSD of PsbH2 with PsbH from two representative non-FaRLiP cyanobacteria. RMSD is reported in units of Å. The FRL-specific structure is in red font.**

|  | **PsbH2 from *Synechococcus* 7335** | **PsbH from *Synechocystis* 6803** | **PsbH from *T. vulcanus*** |
| --- | --- | --- | --- |
| **PsbH2 from *Synechococcus* 7335** | 0.000 | 0.612 | 0.632 |
| **PsbH from *Synechocystis* 6803** | 0.612 | 0.000 | 0.246 |
| **PsbH from *T. vulcanus*** | 0.632 | 0.246 | 0.000 |

**Supporting Data**

**Supplementary Data 1. Jupyter Notebook for the cone scan analysis and cone scan data files (external file).**

**Supplementary Data 2. GitHub link for H-bond search.** This script identifies the Chl binding sites that have possible H-bond donors within a certain radius ('radius') around a pivot ('pivot') atom. Column DN corresponds to the list of atoms that are considered as possible H-bond donors and column AN corresponds to the list of possible H-bond acceptors. There is an option to exclude the backbone amide nitrogen atoms in α-helices ('exclude_alpha_helix') because those atoms are already engaged in H-bonding and therefore do not have a proton to donate otherwise. With the angleT option you may exclude possible H-bond donors that are on the top of the Chl plan, which is defined by the Mg and the N atoms.

[https://github.com/mamin03/Chl-binding](https://nam12.safelinks.protection.outlook.com/?url=https%3A%2F%2Fgithub.com%2Fmamin03%2FChl-binding&data=05|01|christopher.gisriel@yale.edu|d0d61dc33d144e5a457508daac8b46d4|dd8cbebb21394df8b4114e3e87abeb5c|0|0|638012011445507447|Unknown|TWFpbGZsb3d8eyJWIjoiMC4wLjAwMDAiLCJQIjoiV2luMzIiLCJBTiI6Ik1haWwiLCJXVCI6Mn0%3D|3000|||&sdata=%2F8tfqXs0EQH%2BaL4oltSKzAHKhUim%2BtRD544vbOrO6ik%3D&reserved=0)
